# A Physiologically-Based Pharmacokinetic Model of Ruxolitinib and Posaconazole to Predict CYP3A4-Mediated Drug–Drug Interaction Frequently Observed in Graft versus Host Disease Patients

**DOI:** 10.3390/pharmaceutics14122556

**Published:** 2022-11-22

**Authors:** Bettina Gerner, Fatemeh Aghai-Trommeschlaeger, Sabrina Kraus, Götz Ulrich Grigoleit, Sebastian Zimmermann, Max Kurlbaum, Hartwig Klinker, Nora Isberner, Oliver Scherf-Clavel

**Affiliations:** 1 Institute for Pharmacy and Food Chemistry, University of Würzburg, 97074 Würzburg, Germany; 2 Department of Internal Medicine II, University Hospital Würzburg, Oberdürrbacher Strasse 6, 97080 Würzburg, Germany; 3 Core Unit Clinical Mass Spectrometry, Division of Endocrinology and Diabetology, Department of Internal Medicine I, University Hospital Würzburg, 97070 Würzburg, Germany; 4 Faculty of Chemistry, Aalen University, Beethovenstraße 1, 73430 Aalen, Germany

**Keywords:** physiologically based pharmacokinetic (PBPK) modeling, ruxolitinib, posaconazole, drug–drug interactions (DDIs), graft versus host disease, cytochrome P450 3A4 (CYP3A4), pharmacokinetics

## Abstract

Ruxolitinib (RUX) is approved for the treatment of steroid-refractory acute and chronic graft versus host disease (GvHD). It is predominantly metabolized via cytochrome P450 (CYP) 3A4. As patients with GvHD have an increased risk of invasive fungal infections, RUX is frequently combined with posaconazole (POS), a strong CYP3A4 inhibitor. Knowledge of RUX exposure under concomitant POS treatment is scarce and recommendations on dose modifications are inconsistent. A physiologically based pharmacokinetic (PBPK) model was developed to investigate the drug–drug interaction (DDI) between POS and RUX. The predicted RUX exposure was compared to observed concentrations in patients with GvHD in the clinical routine. PBPK models for RUX and POS were independently set up using PK-Sim^®^ Version 11. Plasma concentration-time profiles were described successfully and all predicted area under the curve (AUC) values were within 2-fold of the observed values. The increase in RUX exposure was predicted with a DDI ratio of 1.21 (C_max_) and 1.59 (AUC). Standard dosing in patients with GvHD led to higher RUX exposure than expected, suggesting further dose reduction if combined with POS. The developed model can serve as a starting point for further simulations of the implemented DDI and can be extended to further perpetrators of CYP-mediated PK-DDIs or disease-specific physiological changes.

## 1. Introduction

Ruxolitinib (RUX) is an orally administered multi-kinase inhibitor with potent and selective inhibitory activity against Janus-associated kinases (JAK) 1 and 2 and is approved for the treatment of myelofibrosis and polycythemia vera. In 2019 and 2021, the U.S. Food and Drug Administration (FDA) extended the indication to the treatment of steroid-refractory acute and chronic graft versus host disease (GvHD), respectively. The European Medicines Agency (EMA) approved RUX for the treatment of GvHD in March 2022. GvHD is the most common life-threatening complication after allogeneic hematopoietic stem cell transplantation (allo-HSCT) and a challenge to successful transplant outcomes [1,2]. Acute GvHD mainly affects the skin, liver, and gastrointestinal tract. Despite prophylaxis with immunosuppressive agents, about half of the patients undergoing allo-HSCT develop acute GvHD and 30–70% develop chronic GvHD [1,3,4,5]. For first-line treatment of moderate to severe acute and chronic GvHD, systemically high-dosed glucocorticoids are used [6]. However, less than half of the patients treated for acute and only 40–50% of patients treated for chronic GvHD respond to the treatment, respectively [1].

Invasive fungal infections play a major role regarding mortality and morbidity after allo-HSCT [7]. Therefore, antifungal primary prophylaxis is crucial to improve outcomes. Posaconazole (POS) has been shown to be superior to fluconazole for antifungal prophylaxis in GvHD patients and is therefore frequently used [8,9]. It is a potent inhibitor of cytochrome P450 (CYP) 3A4 (IC_50_ = 1.5 µM) and can lead to a strong increase in the exposure of CYP3A4 substrates [10,11,12]. POS is classified a Biopharmaceutical Classification System (BCS) Class II compound with solubility-limited pharmacokinetics and slow absorption and distribution. Oral bioavailability of POS is highly dependent on the formulation.

RUX is a BCS Class I compound, characterized by high permeability, high solubility, and rapid dissolution. RUX is metabolized by CYP3A4 (>50%) and to a lower extent by CYP2C9. Consequently, RUX elimination is susceptible to drug–drug interactions (DDI) if co-administered with POS, potentially increasing RUX exposure. Very common adverse events of RUX are blood- (e.g., anemia, thrombocytopenia, neutropenia) and lymphatic system disorders. In addition, JAK inhibition increases the probability of invasive fungal infections by impacting immune cells (e.g., dendritic cells, T cells), thus contributing to immunosuppression [13,14]. RUX is commonly dosed 10 mg twice daily (BID) in GvHD patients. Due to the high probability of concomitant administration of strong CY3A4 inhibitors (e.g., calcineurin inhibitors, azoles) a lower RUX dose compared to myelofibrosis treatment was chosen for the pivotal studies in GvHD patients without a formal dose-finding study [15]. Dose adjustment is recommended for safety reasons and is based on platelet count, absolute neutrophil count, and total bilirubin elevation. Increased RUX plasma levels due to CYP3A4 or dual CYP2C9/3A4-inhibition may lead to a higher occurrence of adverse events, which is an additional burden in the vulnerable population of GvHD patients.

However, different recommendations regarding dose adjustments for the combination of RUX with strong CYP3A4 inhibitors (e.g., ketoconazole, POS) or dual CYP2C9/3A4 inhibitors (fluconazole) are given by the FDA and EMA. Based on studies with the FDA index inhibitors ketoconazole and fluconazole, the EMA summary of product characteristics (SmPC) recommends a general dose reduction of the RUX single daily dose by 50% with concurrent strong CYP3A4 inhibitors or dual CYP2C9/3A4 inhibitors, regardless of the indication [15,16,17]. In contrast, the FDA label distinguishes between myelofibrosis, polycythemia vera, and GvHD. For GvHD patients, a reduced RUX starting dose of 5 mg BID is only recommended if it is co-administered with fluconazole, whereas dose adjustments when used concomitantly with strong CYP3A4 inhibitors are explicitly excluded. So far, no investigations have been conducted of the combination of RUX and POS.

According to FDA and EMA, physiologically based modeling (PBPK) is a powerful tool to qualitatively and quantitatively analyze the impact of DDI and can be used in lieu of clinical studies [16,18,19,20,21]. In recent years, drug submissions containing PBPK analyses to investigate DDI have significantly increased (to 60%) [20,22]. In general, FDA analysis of regulatory submissions shows that the PBPK model approach has good performance in predicting the effect of CYP3A4 inhibition on the pharmacokinetics of drug substrates [20,21,23]. So far, PBPK modeling is not applied in the clinical routine even though it can be used to guide dose adjustment by predicting the potential DDI of concurrently administered perpetrators and victims. As no investigations have been conducted of the combination of RUX and POS so far, the aim of this work was to develop a PBPK model to describe changes in RUX exposure due to CYP3A4-inhibition by POS. The developed DDI model was used to compare simulated RUX exposure in healthy individuals to observed concentrations in patients treated for aGvHD or cGvHD in the routine clinical setting.

## 2. Materials and Methods

### 2.1. Software

Version 11 of the freely available software PK-Sim^®^, which is part of the Open Systems Pharmacology Suite (Bayer Technology Services, Leverkusen, Germany) [24], was used for POS and RUX model building. Parameter optimization using the integrated Monte Carlo algorithm and sensitivity analyses were also performed with PK-Sim^®^. Extraction of clinical study data from published literature was conducted with the semi-automated tool WebPlotDigitizer (Version 4.3, Ankit Rohatgi, Pacifica, CA, USA). For plot generation, R Studio Version 1.1.383 (RStudio Incorporation, Boston, MA, USA) running R version 3.6.3 (R Foundation for Statistical Computing, Vienna, Austria, 2020) [25] was used. All statistical calculations and investigation of model performance were carried out with Microsoft Excel 2016 Version 16.0 (Microsoft Corporation, Redmond, WA, USA).

### 2.2. Posaconazole Model Development

An extensive literature research was conducted to obtain physicochemical properties (e.g., molecular weight, lipophilicity, pKa, solubility at different pH values) of POS. Human intravenous (i.v.) data from five clinical studies comprising different dose regimens (50 mg, 100 mg, 200 mg, 250 mg, and 300 mg) were used for the development of the initial model [26]. Different methods for the calculation of tissue distribution and cellular permeability were evaluated and values obtained from literature for lipophilicity (LogP) [27], fraction unbound in plasma(fu_p_) [28], and for the catalytic rate constant (kcat) for UGT1A4 [29] were integrated and optimized in a stepwise approach, if necessary. In addition, the influence of glomerular filtration rate (GFR) fraction and biliary clearance was investigated. The model was tested with human i.v. data from a 300 mg single dose (SD) clinical study [30]. POS is available as suspension (SUS) (40 mg/mL), delayed-release SUS (30 mg/mL), delayed release tablet (DR-tablet) (100 mg per tablet) and i.v. formulation (18 mg/mL). The delayed-release SUS was approved only recently (Noxafil^®^ 300 mg PowderMix, FDA: May 2021 and EMA: January 2022) and was therefore not used for model building. As the DR-tablet is less susceptible to changes in gastric conditions or pH-dependent precipitation, two different formulations were modeled for the POS SUS and DR-tablet [31]. The DR-tablet was modeled using concentration-time data from two clinical studies [32,33]. Optimized parameters consisted of intestinal solubility, specific intestinal permeability, and the formulation (time to 50% dissolution, lag time, and dissolution profile). To account for the higher POS exposure after administration of the DR-tablet, the intestinal solubility was adapted in all tablet simulations and fitted to the physiological pH changes along the gastrointestinal tract [34]. POS SUS was modeled using training datasets from two SD [35,36] clinical studies and one multiple dose (MD) [35] clinical study. Particle dissolution was used to model POS SUS. Specific intestinal permeability, solubility at different pH values, and gain per charge were obtained from literature and optimized accordingly to better fit the data. For POS SUS, supersaturation was enabled to account for the precipitation behavior of POS upon entering the small intestine [27]. The precipitated drug was treated as “soluble” so that the precipitated POS amount was added to the solid drug mass. Thereby, the solid fraction available for dissolution and the particle size were increased and the poor solubility in the intestines was modeled more accurately. The thickness of the unstirred water layer and the particle radius were optimized to better simulate the poor solubility of POS SUS in the intestinal pH environment.

**Figure 1:**
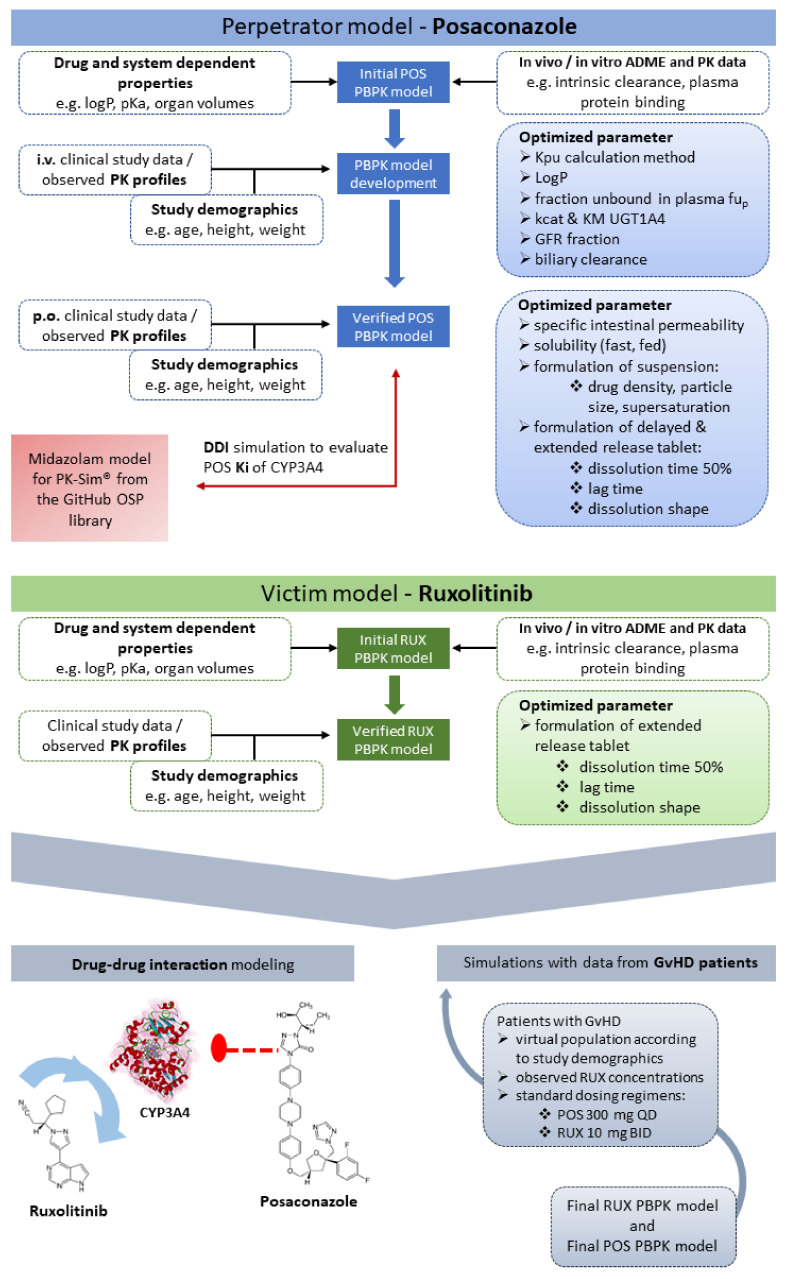
Schematic workflow showing the development and evaluation of the perpetrator (POS) and victim (RUX) PBPK models, which were developed separately. Initial model development started with basic input parameters from the literature. Parameters optimized during model building are shown in the blue and green rectangles, respectively. The final input parameters are given in Tables S2 and S6. The entire model building and evaluation process was supported using clinical study data, used either as training or test dataset (see Tables S1 and S5 for allocation). The final POS and RUX models were applied to simulate DDI between these substances and simulations with data obtained from GvHD patients were conducted (see gray rectangle).

The parameters were tested using SD and MD data from clinical studies [32,35,37], as shown in Table S1. Virtual mean human individuals were created according to the demographics of the respective studies and used in the simulations. A summary of each study regarding the demographics, administration protocols, and their assignment to training or test datasets is documented in Table S1 of the Electronic Supplementary Materials (ESM). A schematic workflow of the POS model development and evaluation including optimized parameters is shown in Figure 1.

### 2.3. Ruxolitinib Model Development

Physicochemical data of RUX obtained by extensive literature search were used as initial input parameters. The RUX model was developed based on eleven datasets from three clinical studies, covering a dose range from 10 to 100 mg. In these studies, RUX was administered orally in different dose regimens (QD, BID, TID), in single or multiple dose scenarios. Four SD clinical studies were used as training datasets [38]. Formulation-related parameters were optimized to model the extended-release tablet (time to 50% dissolution, lag time, and shape of dissolution profile) (Figure 1). The model was tested using data from two SD [39] and five MD clinical studies [40]. A summary of each study regarding the demographics, administration protocols, and the allocation to either the training or the test dataset is given in Table S5 of the ESM.

### 2.4. Model Evaluation

RUX and POS PBPK models were evaluated using various methods according to the guidelines on the reporting of PBPK modeling by the EMA and FDA [19,41]. For a first visual interpretation of the model performance, the trajectories of the predicted plasma concentrations were compared to the respective observed profiles. Goodness-of-fit plots were generated in which the predicted and observed plasma concentrations, predicted and observed maximum concentrations (C_max_), as well as the predicted areas under the systemic drug concentration–time curve from time zero to the time of the last concentration (AUC_last_) were compared. Prediction error (PE) (Equation (1)), mean prediction error (MPE) (Equation (2)) and mean absolute prediction error (MAPE) (Equation (3)) were calculated to evaluate model accuracy and precision.

(1) $$\mathrm{PE}\;\left[\%\right]=\frac{Cpredicted,i-Cobserved,i}{Cobserved,i}\times 100\%$$

(2) $$\mathrm{MPE}\;\left[\%\right]=\frac{1}{n}\times \overset{n}{\underset{i=1}{\sum }}PEi$$

(3) $$\mathrm{MAPE}\;\left[\%\right]=\frac{1}{n}\times \overset{n}{\underset{i=1}{\sum }}\left|PEi\right|$$

Mean relative deviation (MRD, Equation (4)), defined as the average distance of the observed plasma concentration values from the predicted values on a logarithmic scale [42], was calculated for a quantitative measure of model performance. MRD values ≤ 2 were considered acceptable and characterize an adequate model performance in the case that the average of the predicted values was equal to or less than a factor of 2 of the observed values.

(4) $$\mathrm{MRD}=10^{x};x=\sqrt{\frac{1}{n}\overset{n}{\underset{i=1}{\sum }}{\left(log_{10}c_{predicted,i}-log_{10}c_{observed,i}\right)}^{2}}$$

Abbreviations in Equations (1)–(4) are as follows: *c_predicted,i_* = predicted plasma concentration, *c_observed,i_* = corresponding observed plasma concentration, *n* = number of observed values.

Local sensitivity analysis of single parameters of the POS and RUX models was performed, measured as relative changes of AUC_last_ or C_max_. The sensitivity analysis of parameters that had been optimized or might have had a strong influence on the models due to the calculation methods in PK-Sim^®^ was conducted with a variation range of 10.0 and a maximum number of 9 steps.

### 2.5. Drug–Drug Interaction between Posaconazole and Midazolam

To evaluate the inhibitory constant (Ki) of POS for the competitive inhibition of CYP3A4, the developed POS model was combined with a published and evaluated PBPK model for midazolam (MDZ) [43]. Plasma-concentration time profiles from a phase 1 interaction study after i.v. and oral administration of MDZ either given alone or in combination with 200 mg or 400 mg oral POS were used to investigate the effect of oral POS on MDZ exposure. The clinical studies used for DDI modeling of POS and MDZ are summarized in Table S9. The quality of the DDI interaction modeling was evaluated in a stepwise approach. First, the respective plasma concentration-time profiles were visually compared. For a quantitative evaluation, the ratios of AUC_last_ and C_max_ for the administration of i.v. and oral MDZ alone or together with its perpetrator, respectively, were calculated according to Equations (5) and (6). The calculated DDI ratios were compared in each case with the respective values reported by Krishna et al. [44].

(5) $$\mathrm{DDI}\;\mathrm{AU}C_{\mathrm{last}}\;\mathrm{ratio}=\frac{\mathrm{AU}C_{\mathrm{last}}\;\mathrm{MDZ}\;\mathrm{in}\;\mathrm{combination}\;\mathrm{with}\;\mathrm{POS}}{\mathrm{AU}C_{\mathrm{last}}\;\mathrm{MDZ}\;\mathrm{alone}}$$

(6) $$\mathrm{DDI}\;C_{\max}\;\mathrm{ratio}=\frac{C_{\max}\;\mathrm{MDZ}\;\mathrm{in}\;\mathrm{combination}\;\mathrm{with}\;\mathrm{POS}}{C_{\max}\;\mathrm{MDZ}\;\mathrm{alone}}$$

### 2.6. Simulations in Graft versus Host Disease Patients

We used 278 serum samples from 30 patients with either acute or chronic GvHD receiving any dose regimen of RUX collected between February 2019 and February 2021 at the University Hospital of Würzburg as part of a non-interventional prospective clinical trial. The study was approved by the Ethics Commission of the University of Würzburg (ref 199/18-am). All performed procedures were in accordance with the Declaration of Helsinki. Written informed consent was obtained from all patients. RUX dosage, time of last intake, time of sampling, and additional co-medications were recorded during the study. POS dosage was documented during data collection, yet the time of last intake was missing. It was assumed that POS was taken at the same time as RUX and that the concentrations were obtained in a steady state. RUX and POS concentrations were measured in human serum using previously published liquid chromatography methods [45,46]. As the PBPK model was developed and evaluated for interaction between POS and RUX, the dataset was filtered for patients receiving POS as antifungal prophylaxis. Moreover, patients receiving RUX without any strong or moderate CYP3A4 inhibitor were filtered, and simulations for patients receiving RUX with and without its perpetrator POS were conducted separately. One hundred and sixty-three of the RUX concentrations from 19 patients were obtained under concomitant POS administration; 27 RUX concentrations from 7 patients were obtained without the co-administration of POS. All simulations were based on the standard daily RUX dose of 20 mg daily (10 mg BID). For all simulations in GvHD patients, a virtual population (*n* = 100) according to the study demographics was built. Information on the baseline patient demographics can be found in Table S10.

## 3. Results

### 3.1. Posaconazole PBPK Model Building and Evaluation

The best results were obtained for a combination of the Poulin and Theil distribution methods and PK-Sim^®^ standards for distribution method and cellular permeability, respectively. Based on the i.v. data, values for fu_p_, lipophilicity, the katalytic rate constant for UGT1A4, and the specific biliary clearance were optimized to get a better fit of predicted versus observed concentrations. The i.v. model was extended to include oral data after administration of POS suspension (SUS) and delayed release tablet (DR-tablet). The specific permeability calculated based on molecular weight and lipophilicity of the substance within the PK-Sim^®^ software (1.81 × 10^−4^ cm/min) was further optimized in fasted and fed state (5.05 × 10^−5^ cm/min) and used for SUS simulations [47,48]. High-fat or non-fat meal events were modeled with 841 kilo calories (kcal) or 200 kcal, as reported by Courtney et al. [37].

For the DR-tablet simulations, the specific permeability was 4.80 × 10^−5^ cm/s. POS SUS was modeled using particle dissolution with supersaturation enabled and the DR-tablet was modeled using the Weibull function. Particle radius (1.9 µm), thickness of unstirred water (140 µm), dissolution time (145 min), lag time (30 min), dissolution shape (1.67), and drug density (0.37 g/cm^3^) were estimated using parameter identification or improved by manual adjustment. The final parameters used for the POS PBPK model are shown in Table S2.

The final PBPK model was successfully used to describe the observed POS plasma concentrations after single and multiple dose administration of i.v. or oral POS. Simulated plasma profile trajectories were in close concordance with observed data in fasted as well as in fed state. Linear and semilogarithmic plots of predicted versus observed plasma concentration-time profiles after i.v., DR-tablet, or SUS administration are shown in Figure S5a–c and Figure S6a–c, respectively. The model slightly overpredicted low POS plasma concentrations at later times after dosing (see goodness-of-fit plot, Figure S1); 90.44% of all simulated plasma concentrations fell within 2-fold of the corresponding concentrations observed. Separate goodness-of-fit plots for i.v., DR-tablet, and SUS can be found in Figure S2a–c. All predicted AUC_last_ and 95% of the predicted C_max_ values were within the 2-fold acceptance criterion (Figure S3).

For the i.v. simulation, a MRD of all predicted plasma concentrations ≤2.0 was achieved in all simulations (MRD range 1.42–1.75) and 87.3% of all simulated plasma concentrations were within 2-fold of the corresponding concentrations observed. The MPE range was −29.65% to 39.60% and the MAPE range was 25.01% to 46.50% (Table S4). Oral simulations of different dosing regimens either in fasted or fed state after DR-tablet or SUS administration had a MRD of 1.52 (1.19–2.36) with 12/13 simulations ≤ 2.0, while 91.72% of simulated plasma concentrations after SUS administration and 91.67% after DR-tablet administration were within 2-fold of the corresponding concentration observed. Ratios for predicted versus observed values were 0.66 to 1.44 (AUC_last_) and 0.55 to 2.26 (C_max_) (Table S3). The local sensitivity analysis revealed that variation in lipophilicity had the greatest impact on changes in AUC_last_ after a 100 mg POS single tablet administration, followed by fu_p_ (Figure S4).

### 3.2. Ruxolitinib PBPK Model Building and Evaluation

For the RUX PBPK model, several calculation methods for tissue distribution and cellular permeabilities were tested during model development. The best results were obtained for the Rodgers and Rowland tissue distribution [49,50] in combination with the PK-Sim^®^ standard method for the calculation of cellular permeabilities. Drug-dependent parameters such as lipophilicity, solubility, and fu_p_ found in the literature were appropriate and not adapted. According to Umehara et al., biliary and renal excretion are negligible and were therefore not implemented into our model [51]. CYP2C9 and CYP3A4 metabolizing enzymes were included and a first order process for the metabolic enzyme activity was chosen, which fully covered RUX elimination. The specific clearance used in the simulations was 0.65 L/µmol/min for the CYP2C9 process and 0.46 L/µmol/min for the CYP3A4 process and was calculated based on the in vitro intrinsic clearance by Umehara et al. [51]. The integrated Weibull function was used to create an extended-release tablet and parameters for the dissolution time (15.0 min) and the dissolution shape (1.10) were adapted to fit the observed data. The best results were obtained for a dissolution time of 15 min and a dissolution shape of 1.10. All input parameters used in the final RUX PBPK model can be found in Table S6.

**Figure 2:**
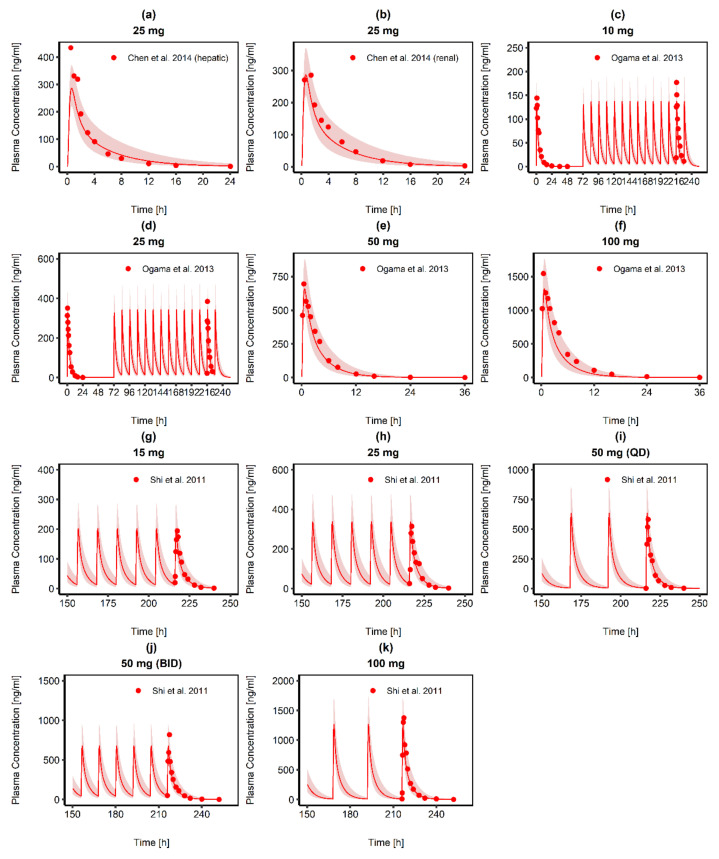
Predicted RUX plasma concentration-time curve profiles (solid red lines) and observed RUX concentrations (red dots), obtained after single (**a**,**b**,**e**,**f**) and multiple RUX tablet (**c**,**d**,**g**–**k**) administration. The red shaded area represents the predicted population geometric standard deviation in each case. The ESM contains detailed information about the study protocols and RUX model performance for each simulation. QD: once daily, BID: twice daily. Source: [38,39,40].

The final RUX PBPK model successfully predicted observed RUX plasma concentrations after single as well as multiple dosing in the investigated populations (see Figure 2 for linear plots and Figure S11 for semilogarithmic plots) and 86.79% of the observed data fell within 2-fold of the corresponding concentration observed (Figure S8). Ratios for predicted versus observed values were 0.68 to 1.12 (AUC_last_) and 0.65 to 1.04 (C_max_) (Table S7). The model slightly underpredicted RUX exposure, as 9/11 ratios for predicted versus observed AUC_last_ were <1. This effect was less distinct for C_max_. Except for three simulations, all predicted C_max_ values were within +/−10% compared to the observed C_max_. However, all predicted AUC_last_ and C_max_ values fell within the 2-fold acceptance criterion (Figure S9). The mean MRD was 1.58 (1.20 to 2.23) and 9/11 simulations fulfilled the acceptance criterion. All values for MRD, MPE, and MAPE are listed in Table S8. Local sensitivity analysis showed that the RUX model was most sensitive to fu_p_ followed by lipophilicity (see Figure S10).

### 3.3. Drug–Drug Interaction Modeling

#### 3.3.1. Posaconazole and Midazolam

**Figure 3:**
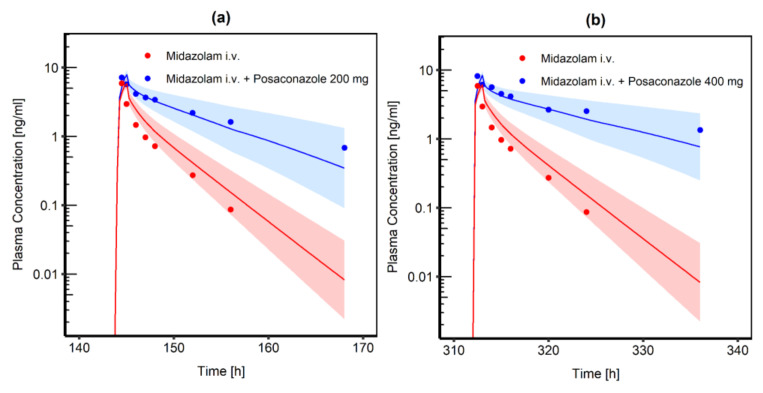
Comparison of simulated MDZ venous blood plasma concentration-time profiles (semilogarithmic) following i.v. administration of 0.4 mg MDZ infusion. The blue shaded area represents the geometric mean SD for population simulations of the resulting MDZ plasma concentrations if co-administered with either (**a**) 200 mg oral POS or (**b**) 400 mg oral POS and an inhibitory constant Ki = 5.22 nmol/L for the CYP3A4 interaction process. The red shaded area represents the geometric mean SD for population simulations without concomitant POS administration. Geometric means are shown as a blue line (with POS administration) or a red line (without POS administration). Observed data are shown as blue and red dots, respectively.

A competitive inhibition process was assumed for the interaction of POS and MDZ with CYP3A4. POS reversibly binds to CYP3A4 and competes with MDZ for free binding sites. All input parameters of the POS and MDZ model were transferred, expect for Ki, which was estimated. The Ki start value was taken from the literature (0.42 µM) [52]. Based on the 200 mg and 400 mg MDZ i.v. administration, Ki parameter estimation led to an optimized value of 5.22 × 10^−3^ µM. Comparison of 200 mg and 400 mg i.v. MDZ administration with and without concomitant POS administration can be found in Figure 3. Calculated DDI ratios for C_max_ and AUC_last_ were comparable to the ratios calculated from the respective clinical study by Krishna et al. [44] (Table 1). As Ki was estimated based on MDZ i.v. administration, as a proof-of-concept, DDI ratios were calculated for data after oral MDZ administration and were comparable to the reported values (Table 1).

#### 3.3.2. Posaconazole and Ruxolitinib

The resulting Ki for the inhibition of CYP3A4 was transferred to the POS model and the model was combined with the developed RUX model for DDI simulations. Simulations with a virtual population receiving 300 mg oral POS DR-tablet and 10 mg BID RUX were conducted and simulated C_max_ for RUX without POS co-administration was 116.31 ng/mL. The median C_max_ for the simulation of RUX plasma concentration if given together with its perpetrator was 20.5% higher (C_max_ = 140.21 ng/mL). Simulated RUX exposure was about 59% higher if co-administered with POS (AUC_last_ = 382.17 ng·h/mL) compared to RUX administration alone (AUC_last_ = 239.88 ng·h/mL). The calculated DDI ratio was 1.21 for C_max_ and 1.59 for AUC_last_, respectively.

### 3.4. Simulation of Graft versus Host Disease Patients

**Figure 4:**
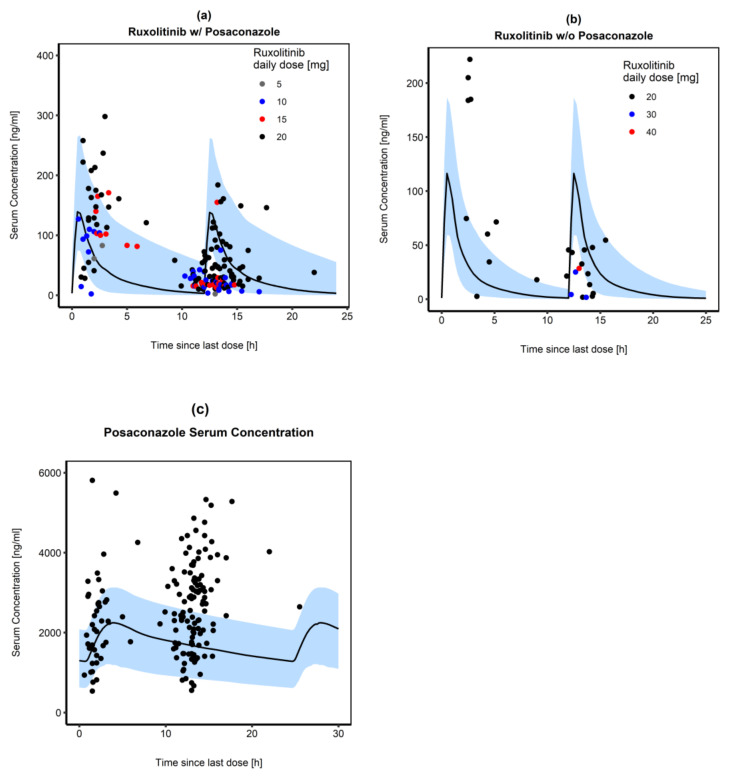
Comparison of simulated RUX serum concentration-time profiles following oral administration (10 mg BID) if (**a**) co-administered with 300 mg oral POS and (**b**) given without its perpetrator. Observed RUX concentrations are shown as dots with different colors, representing different RUX daily doses actually taken by the patients. Median RUX concentrations are shown as a black line in each part. Part (**c**) represents predicted (black solid line) and observed POS concentrations (black dots) after administration of 300 mg oral POS tablet once daily. The blue shaded areas represent the 5% to 95% prediction intervals for population simulations (*n* = 100) of the resulting RUX and POS plasma concentrations, respectively.

In the GvHD study population, a high interindividual variability in observed RUX concentrations was seen, especially shortly after tablet intake (within five hours after RUX administration). We found 64.42% of the observed serum concentrations after 10 mg BID RUX administration in combination with POS were within the 5% to 95% prediction intervals of the corresponding simulation (Figure 4a). Even without concomitant administration of oral POS, model-predicted median RUX serum concentration for the administration of 10 mg RUX BID was lower than the serum concentrations observed in the GvHD patients of the clinical study (Figure 4b). The POS model predicted trough concentrations of 1282.16 ng/mL and maximum plasma concentrations of 2245.70 ng/mL (300 mg QD POS DR-tablet, simulated for day 10), while measured concentrations revealed a median concentration of 2392 ng/mL (range: 21–5808 ng/mL, *n* = 169, 300 mg QD POS DR-tablet) and 2441 ng/mL (range: 1854–2784 ng/mL, *n* = 8, 200 mg TID POS SUS) [53]. About half (51.25%) of the observed POS values fell within the 5% to 95% prediction interval of the simulation and 43.75% of the observed POS concentrations were above the predicted range. Simulated and observed POS serum concentrations are displayed in Figure 4c.

## 4. Discussion

To the best of our knowledge, this work presents the first PBPK models for POS and RUX using PK-Sim^®^ developed for application in the clinical routine. Seven PBPK models using different PBPK software (SimCYP^®^ (Certara Holdings Ltd., Sheffield, UK) or GastroPlus (SimulationsPlus, Lancaster, CA, USA) have been published for POS [27,54,55,56,57,58,59]. Only three were developed to model potential DDI with one being developed for application to the clinical routine. The remaining models were developed for the assessment of bioequivalence or to describe POS behavior in the gastrointestinal (GI) tract. In the literature, feasibility of the PBPK approach has been shown for RUX and the index inhibitors ketoconazole and fluconazole using SimCYP^®^, and the findings were compared to clinically performed DDI studies as a proof-of-concept to support regulatory submissions [60,61]. However, no investigations on the combination of RUX with POS have been conducted so far; yet this is of significant clinical interest because POS is frequently co-administered with RUX in patients with aGvHD and cGvHD [9].

The developed PBPK DDI model predicted an increase in RUX C_max_ and AUC_last_ by 20.5% and 59%, respectively, due to the concomitant POS administration. Using the web-based DDI predictor (https://www.ddi-predictor.org/, accessed on 9 September 2022) with RUX as substrate and POS 300 mg daily as interactor, an increase in AUC ratio of 1.35 was estimated (95% prediction interval 0.96–1.89). Our AUC_last_ ratio of 1.59 is within the proposed prediction interval.

Using measured RUX and POS concentrations in our study population, we were able to evaluate the predicted concentrations, and 64.42% of the observed RUX concentrations (Figure 4a) and 51.25% of the observed POS concentrations (Figure 4c) were within the prediction intervals. Approximately one-third of the observed values lay outside the prediction intervals for two main reasons. As we used data obtained in daily clinical routines, it was not 100% certain whether all concentrations were really trough levels or whether a new dose had already been taken, as is simulated in Figure 4a. This was especially the case for concentrations observed between 12 and 15 h since the last dose. Secondly, we would like to mention the following: even though the PBPK model included the potential DDI, a greater proportion of the observed RUX concentrations in patients receiving 10 mg RUX BID concomitantly with POS were above the predicted median RUX concentration. The observed data are real-life data and were not obtained within a controlled DDI study. Thus, observed concentrations were not only influenced by the DDI between RUX and POS but also by the complex disease, comorbidities, and numerous further medications taken by the patients. Isberner et al. reported a higher RUX exposure in GvHD patients compared to myelofibrosis patients, which was attributed to a lower clearance, which was also reported by Chen et al. (50% and 66.7%, respectively) [53,62]. Isberner et al. allocated the reduced clearance to further DDI caused by a combination of several moderate and weak CYP3A4 or CYP2C9 inhibitors (e.g., atorvastatin and amiodarone) and changes in hepatic clearance due to liver dysfunction, which is, however, hypothetical.

They also observed an additional reduction of RUX clearance by 15% due to comedication with at least one strong CYP3A4 inhibitor, suggesting that in aGvHD and cGvHD patient dose modification may be necessary [53]. This is in accordance with our findings, as concentrations obtained from patients receiving a lower RUX dose (5 mg QD, BID and TID, respectively) were significantly closer to the predicted median RUX concentration (Figure 4a) and within the 5% to 95% prediction intervals of the model simulation in healthy individuals (10 mg BID).

Thus, the explicit exclusion of dose reduction in aGvHD and cGvHD patients recommended by the FDA seems to lead to overexposure in a considerable proportion of patients. It may be for this reason that the EMA advises a general dose reduction by approximately 50% of the unit RUX dose if co-administered with strong CYP3A4 inhibitors such as ketoconazole and POS or dual CYP2C9/3A4 inhibitors such as fluconazole. In the EMA SmPC, ketoconazole and POS are both listed as strong CYP3A4 inhibitors without consideration of their exact inhibitory potency towards CYP3A4. However, our study showed that POS has a lower impact on RUX exposure compared to ketoconazole (C_max_ and AUC_last_ 33% and 91%, respectively) [61] and fluconazole (C_max_ and AUC_last_ 47% and 234%) [60], suggesting that a 50% unit dose adjustment may not be appropriate in general for the azoles mentioned and dose modifications should also be adapted to the individual patient, according to his or her further medication and etiopathology.

Taken together, observed concentrations outside the prediction intervals are likely due to imprecisions in the measured values, due to limitations within the model, and due to the physiological specifics of GvHD patients. Deviations between predicted and observed RUX concentrations in the clinical routine may be caused by the study design because time of last dose intake and time of sampling were reported by the patient and the nurse, respectively, which is susceptible to bias. In addition, the primarily aim of the conducted study was to observe RUX concentrations in GvHD patients and measurement of POS concentrations was added by amendment. Therefore, it is not fully clear when POS was taken with respect to sampling. The best approximation was the assumption that POS tablet or SUS were taken at the same time as RUX, which may not be true for all events. In addition, PK studies used to obtain observed concentrations for POS and RUX model development did not contain raw data. A digitizing software was used, which is a common procedure, yet it is a source of potential imprecision. Further, allo-HSCT patients receive extensive co-medication and often have renal or hepatic impairments resulting from chemotherapy, radiation, or GvHD. GvHD also affects the GI mucosa, which may affect the absorption processes, leading to the observed variabilities in the POS and RUX exposure. In addition, the underlying disease and patients are heterogenous. So far, no quantitative disease model for GvHD exists, which is why the specific physiological alterations of these patients could not be quantitatively included in the model. Therefore, a healthy population using the demographics of the GvHD study population was used for the simulations in PK-Sim^®^. However, this population does not represent every individual and the full complexity of the disease and may explain observed deviations. This approach was nevertheless chosen, as in vivo DDI studies are usually conducted in healthy individuals and, overall, the model is appropriate to predict the magnitude of DDI. As soon as more precise and quantitative knowledge about the underlying disease-specific physiological alterations is available, physiological parameters within the PBPK model can be adapted, which is a considerable benefit of PBPK modeling.

The final RUX PBPK model is characterized by good model performance, as demonstrated by comparison of predicted to observed plasma concentration-time profiles and the respective goodness-of-fit plots, the calculation of MRD values, as well as the comparison of predicted to observed AUC_last_ and C_max_ values (ESM Figures S8, S9 and S11 and Tables S7 and S8). To simulate DDI between RUX and POS, the Ki value for CYP3A4 inhibition by POS was successfully evaluated using a previously published MDZ model. The initial Ki value of POS found in literature was too high as it underpredicted the CYP inhibition process and consequently MDZ plasma concentrations. The optimized value (5.22 × 10^−3^ µM) was in accordance with the Ki value optimized by Bhantnagar et al. (5.5 × 10^−3^ µM) and in line with in vivo findings by Clearly and colleagues (5 × 10^−3^ µM) [52,54,59].

POS model development was challenging, as in general, in silico prediction of in vivo release and exposure of BCS class II compounds and weak bases is not trivial because the in vivo drug dissolution is highly dependent on the GI physiology (e.g., bile component, amount of fluid, and pH in the GI section), which is dynamic and subject to immense inter- and intra-individual fluctuations [28,63]. The lack of published in-house data from preclinical drug development, sparse data on formulation-specific properties, missing information about the patients and the underlying diseases additionally hampered model building [22]. Nonetheless, POS SUS and the commonly used DR-tablet were successfully integrated in the model, so that the model was appropriate to describe POS exposure. Parameter identification and manual optimization were helpful to appropriately fit concentration-time data to formulation-related parameters so that observed concentration-time profiles were within the prediction intervals. Tissue distribution and cellular permeability logP, fu, kcat for UGT1A4, GFR fraction, and biliary clearance, which describe the PK after the absorption, were successfully described based on i.v. data only, so that inaccuracies and bias from drug absorption processes were mostly eliminated.

A high interindividual variability was observed in the measured POS concentrations but they are in accordance with values found in the literature. Cornely et al. reported a mean average concentration of 2370 ng/mL (range: 680–9520 ng/mL, once daily, 3 × 100 mg, DR-tablet, *n* = 210), a mean minimum plasma concentration of 2110 ng/mL (range: 445–9140 ng/mL, once daily, 3 × 100 mg DR-tablet, *n* = 210), and a mean maximum plasma concentration of 2390 ng/mL (coefficient of variation (CV) 43%, once daily, 3 × 100 mg, DR-tablet, *n* = 210) in allo-HSCT recipients [64]. Krishna et al., reported a mean average plasma concentration of 1310 ng/mL (CV 31%, 200 mg, once daily, *n* = 8, measured on day 14) and 2550 ng/mL (CV 38%, 200 mg twice daily, *n* = 8, measured on day 22), and 2360 ng/mL (CV 54%, 400 mg once daily, *n* = 8, measured on day 14) taking the POS DR-tablet. The observed variability in POS PK probably contributes to the observed variability in RUX PK and depending on the POS concentration, the interaction may be more or less pronounced. Based on the model, different tested POS dosage (150 mg, 300 mg, 600 mg QD) resulted in different RUX exposure (337.25, 356.51, 367.70 ng · h/mL, respectively), which is consistent with the underlying mechanism. According to the saturable mechanism of CYP inhibition, no linear but rather a saturable increase in RUX exposure can be assumed.

POS free-base has a weak basicity and is well soluble at a low pH and less soluble at a higher pH (e.g., at fasted state stomach (pH 1) 0.79 mg/mL and 0.001 mg/mL at fed state (pH 7.0)), and the absorption of POS is rate-limited by dissolution [27,36,65,66]. After dissolution in the stomach, a substantial amount of dissolved POS precipitates reaching the intestine and is therefore not available for absorption [27]. The systemic exposure of the SUS is highly dependent on food and its fat content [65]. Compared to the fasted state, AUC is four and 2.6 times greater depending on the fat content (50 g and 14 g of fat, respectively). Considering that time and amount of fat content highly impact POS exposure, it can be concluded that fat enhances dissolution of POS in the intestine. This happens either through emulsion or micelle building or due to the increased release of bile salts or lecithin. The changes in the absorption kinetics by an enhanced dissolution through fat could not be fully described by the model, leading to underprediction of the POS absorption after administration of the SUS with a high-fat or a non-fat meal. This may be attributable to the fact that PK-Sim^®^ only allows definition of food events based on the caloric supply and no input of a specific fat content. A high-fat meal can only be added by assuming that for each g fat, 9 kcal are supplied. This is not a true illustration of the food composition and leads to imprecision. Changing the solid fraction of the meal did not improve model fit and was therefore kept at 0.8.

The POS PBPK model showed a steeper absorption phase in fasted state compared to the observed data for dosing at 800 mg and the initial dose of multiple dosing (see Supplementary Materials, semilogarithmic plots for simulations for Ezzet et al.). This may be explained by the fact, that in fasted state, the precipitation kinetics of POS is crucial and accounts for the high intersubject variability of POS plasma concentrations [35]. This is also backed up by the respective study, which showed an intersubject coefficient of variation for the absorption rate constant and bioavailability of 18–70% and 52–73%, respectively. The PBPK model does not properly capture the precipitation in the intestines, which leads to the greater absorption. However, the observed deviation is regarded as neglectable for the intended application of the POS PBPK model because the systemic daily exposure for 200 mg multiple dosing is within the prediction interval and in clinical routine an 800 mg single dose is not applied.

Using the calculated or optimized specific permeability for the DR-tablet formulation led both to underprediction in absorption and systemic exposure. Ultimately, the intestinal permeability was adapted to model the higher bioavailability of the DR-tablet. The used specific permeability of 4.80 × 10^−5^ cm/s is the apparent in vitro CaCo2-cell permeability reported by Hens and colleagues [55]. It is clear that the CaCo-2 cell permeability is not equal to the effective permeability, yet Walraven and colleagues reported an in-house effective permeability of 4.02 × 10^−5^ cm/s, which was also similar to the CaCo2-cell permeability [67]. The higher specific intestinal permeability was sufficient to simulate a faster uptake of POS upon improved intestinal solubility and supersaturation stability when the DR-tablet was used. Using a lower specific intestinal permeability for POS SUS was sufficient to account for the fact that the dissolved amount of POS that can diffuse over the membrane over time is less if POS SUS is used. The lower specific permeability also reflects that POS is not sufficiently released from the SUS formulation and therefore not highly available for transcellular permeation. Bhatnagar and colleagues developed a PBPK model for application in the clinical routine to predict the DDI between Venetoclax and POS using SimCYP^®^. They faced the same issue and were able to solve the discrepancy by adjusting the effective permeability (6.41 × 10^−4^ cm/s), the bile micelle partitioning coefficient, and the intrinsic solubility [54]. Cristofoletti and colleagues developed a PBPK model of POS also using SimCYP^®^ and used 3.7 × 10^−4^ cm/s as effective permeability [57]. The different values show that depending on the software and the studies used for parameter identification, the intestinal permeability may be different and the calculated value using molecular weight and lipophilicity may not be sufficient to predict the observed data. The observed difficulties are in line with the fact that the two formulations are not interchangeable due to differences in PK resulting from differences in solubility and permeability during GI tract passage. Sensitivity analysis further backs up these findings, as lipophilicity has an outstanding impact on AUC_last_ (−6.81).

In summary, prediction of the PK using PK-Sim^®^ for a poor formulation is not straightforward if data concerning the solubility of the compound and the dissolution of the formulation are not available as model input parameters. Even with in vitro dissolution data, the prediction of in vivo dissolution remains challenging because knowledge about the impact of the dynamic GI environment on drug and formulation behavior is scarce [68]. Garcia et al., recently compared the two modeling platforms PK-Sim^®^ and SimCYP^®^, building comprehensive PBPK models for simvastatin [69]. They found major differences in the implementation of absorption models, with lower complexity and flexibility regarding input of formulation and passive permeability in PK-Sim^®^ compared to SimCYP^®^. SimCYP^®^. offers different options for the input of absorption parameters, including built-in correlation methods to scale in vitro measured values as well as the SimCYP^®^ In Vitro data Analysis (SIVA) toolkit [70]. In PK-Sim^®^, on the other hand, Garcia et al. also had to estimate intestinal passive permeability and formulation dissolution parameters based on available clinical study data, as there were no other options.

Expect for one patient, the GvHD study population received the POS DR-tablet and our simulations for that population were done with the DR-tablet accordingly. If POS SUS is administered instead of the DR-tablet, the expected DDI with RUX should be evaluated carefully, as we used different intestinal permeabilities to account for the different formulations. For some study populations, sparse information on the baseline patient demography was available. Only one study reported age, height, weight, and BMI of the study population [30]. One study reported age, height, and weight [33], and five studies reported data for age and either weight, height, or BMI of the study population [26,32,35,36,37]. To build the virtual populations in PK-Sim^®^, the missing demographic data were estimated, which led to imprecisions. In addition, the virtual population generated via the implemented PK-Sim^®^ algorithm differs in some cases from the mean individual used for model building, which influences model precision. As an example, the model was able to precisely predict the plasma concentration-time profile of POS for the mean individual created according to Vuletic et al. [36] and simulated concentrations were in close concordance with the observed data (Figure S7). However, a high bias (MPE = 147.64%), low precision (MAPE = 181.61), and a MRD of 2.36 was obtained in the population simulation with the virtual population (Figure S7).

Summing up, the combination of RUX with POS is of significant clinical relevance as an increase of RUX AUX by 60% is associated with higher probability of adverse events due to RUX overexposure. The other way round, it is also conceivable that too low RUX exposure is achieved if, for example, POS is discontinued or exchanged for a substance that is not a CYP3A4 inhibitor (e.g., Amphotericin B). This could result in therapy failure if the RUX dose is not increased accordingly. Our findings showed that using RUX at a standard dosage, if co-administered with POS in GvHD patients, led to higher exposure compared to simulations in a healthy population. Thus, the FDA recommendation should be considered with caution and patients at risk of RUX overexposure or with a high potential of adverse events occurring should be identified, which can be supported by the developed model. The developed POS and RUX PBPK models can be combined with other existing PBPK models of additional perpetrators or victims in PK-Sim^®^ to describe DDI interaction and applied for dose adjustment in the clinical routine. Future investigations should include the investigation of GvHD-specific physiological alterations, which could be integrated into PBPK models to develop a more accurate GvHD population. In this context, one could also try to distinguish between aGvHD and cGvHD, as the patient populations are often clinically very different, also in terms of co-medication. Additionally, the PBPK models, especially the POS DR-tablet and POS SUS model, should be further validated with measured concentrations from future observational studies.

## 5. Conclusions

Depending on the regulatory authority and the time of approval, different dose recommendations exist for the combination of RUX with strong CYP3A4 inhibitors, which complicates the application in the clinic. For the application in PK-Sim^®^, two separate PBPK models for RUX and POS were successfully set up. The PBPK modeling approach was used to predict a DDI scenario for POS and RUX. RUX plasma exposure simulated with the final DDI model was compared to observed concentrations in patients treated for aGvHD or cGvHD in the routine clinical setting, revealing that standard dosing in these patients may not be adequate and reduced RUX doses should be administered depending on the concomitantly administered azoles and their inhibition potency. Due to the complexity of the disease and intake of extensive co-medication, RUX plasma concentration can be higher than expected. It is therefore advisable to monitor plasma levels and adjust RUX dosing accordingly. The DDI model can be expanded to other perpetrators or victims, e.g., fluconazole and could be further optimized by the implementation of physiological changes in GvHD patients, if these are sufficiently investigated. The model can serve as a starting point to implement PBPK modeling in the clinical routine to predict potential DDI in vulnerable patients and to guide dose adjustment.

## Figures and Tables

**Table 1:** DDI ratio for C_max_ and AUC_last_ calculated for the administration of MDZ together with POS.

POS	MDZ 0.4 mg i.v. SD		MDZ 2.0 mg Oral SD	
DDI Ratio	C_max_	AUC_last_	C_max_	AUC_last_
200 mg oral SUS	1.42 ^a^ *1.30 ^b^*	3.38 ^a^ *4.42 ^b^*	2.74 ^a^ *2.20 ^b^*	5.54 ^a^ *4.99 ^b^*
400 mg oral SUS	1.41 ^a^ *1.68 ^b^*	4.43 ^a^ *6.23 ^b^*	3.02 ^a^ *2.33 ^b^*	6.95 ^a^ *5.26 ^b^*

^a^ DDI ratio for C_max_ and AUC_last_ calculated with MDZ and POS model. ^b^ Values taken from the literature (in vivo) [44].

## Data Availability

All modeling files including utilized clinical study data can be found here: https://github.com/Open-Systems-Pharmacology (accessed on 21 November 2022).

## Supplementary Materials

The following are available online at https://www.mdpi.com/article/10.3390/pharmaceutics14122556/s1, Electronic Supplementary Materials: Additional information on model development and evaluation including Figures S1–S11 and Tables S1–S10.
