# Supplementary material for: A Physiologically-Based Pharmacokinetic Model of Ruxolitinib and Posaconazole to Predict CYP3A4-Mediated Drug–Drug Interaction Frequently Observed in Graft versus Host Disease Patients
Source: Pharmaceutics. 2022 Nov 22;14(12):2556. doi: 10.3390/pharmaceutics14122556 (PMC9785192; doi:10.3390/pharmaceutics14122556)
Supplement: Supplementary file 1 [file pharmaceutics-14-02556-s001.zip › pharmaceutics-2018956-supplementary.pdf]

Pharmaceutics

Electronic Supplementary Material

A physiologically based pharmacokinetic model of Ruxolitinib and Posaconazole to predict CYP3A4 mediated Drug-Drug Interaction frequently observed in Graft versus Host Disease Patients

Bettina Gerner [1], Fatemeh Aghai-Trommeschlaeger [2], Sabrina Kraus [2], Götz Ulrich Grigoleit [2,4], Sebastian Zimmermann [1], Max Kurlbaum [5], Hartwig Klinker [2], Nora Isberner [2], Oliver Scherf-Clavel [1,3*]

1 Institute for Pharmacy and Food Chemistry, University of Würzburg, Germany
2 Department of Internal Medicine II, University Hospital Würzburg, Oberdürrbacher Strasse 6, 97080, Würzburg, Germany
3 Faculty of Chemistry, Aalen University, Beethovenstraße 1, 73430 Aalen, Germany
4 Present Address: Department of Hematology, Oncology and Immunology, Helios Hospital Duisburg, Duisburg, Germany
5 Department of Internal Medicine I and Core Unit Clinical Mass Spectrometry, Division of Endocrinology and Diabetology, University Hospital Würzburg, Würzburg, Germany

*Correspondence: oliver.scherf-clavel@hs-aalen.de; Tel.: +49-7361-5763552

Contents

1 Posaconazole **3**

1.1 Clinical studies **3**

1.2 Drug-dependent parameters **5**

1.3 Model evaluation **6**

1.3.1 Goodness-of-fit plots of predicted vs observed plasma concentrations 6

1.3.2 Goodness of fit plot AUC_{last} and C_{max} 8

1.3.3 Comparison of predicted and observed AUClast and Cmax 9

1.3.4 Bias, prediction and mean relative deviation of plasma predictions 10

1.3.5 Sensitivity analysis 11

1.3.6 Linear plots 12

1.3.7 Semilogarithmic plots 14

1.3.8 Comparison of individual and population simulation 16

2 Ruxolitinib **17**

2.1 Clinical studies **17**

2.2 Drug-dependent parameters **18**

2.3 Model evaluation **19**

2.3.1 Goodness-of-fit plots of predicted vs observed plasma concentrations 19

2.3.2 AUC_{last} and C_{max} goodness-of-fit plots 20

2.3.3 Comparison of predicted and observed AUClast and Cmax 21

2.3.4 Bias, prediction and mean relative deviation of plasma predictions 22

2.3.5 Sensitivity analysis 22

2.3.6 Semilogarithmic plots 23

3 Drug-drug interaction simulation posaconazole and midazolam **24**

3.1 Clinical studies **24**

4 Simulation of graft-versus-host disease patients **25**

5 References **25**

1 Posaconazole

1.1 Clinical studies

All clinical studies used for posaconazole PBPK model building and evaluation are summarized in Table S 1-1

Table S1. Posaconazole clinical study data used for model development and evaluation

Study	Dose [mg]	Treatment	n	Men [%]	Age [yrs]	Weight [kg]	Height [cm]	BMI [kg/m²]	Dataset	References
Kersemaekers et al. (2015)	50	iv, SD (30 min)	72	46	18-65	n.r.	n.r.	19-35	training	[1]
Kersemaekers et al. (2015)	100	iv, SD (30 min)	72	46	18-65	n.r.	n.r.	19-35	training	[1]
Kersemaekers et al. (2015)	200	iv, SD (30 min)	72	46	18-65	n.r.	n.r.	19-35	training	[1]
Kersemaekers et al. (2015)	250	iv, SD (30 min)	72	46	18-65	n.r.	n.r.	19-35	training	[1]
Kersemaekers et al. (2015)	300	iv, SD (30 min)	72	46	18-65	n.r.	n.r.	19-35	training	[1]
Li et al. (2019)	300	iv, SD (30 min)	18	67	32.5 (20-44)	63 (51-76)	166 (149-178)	22.9 (19-24)	test	[2]
Krishna et al. (2012a)	200	po, tab, SD/MD	10	50	47.7 (33-59)	74.85 (61-100)	165.60 (156-175)	n.r.	training	[3]
Krishna et al. (2012a)	400	po, tab, SD/MD	9	67	43.8 (31-56)	72.89 (61-86)	168.78 (155-181)	n.r.	test	[3]
Krishna et al. (2012b)	100	po, tab, SD (fasting)	16	50	31.4 (19-45)	n.r.	n.r.	26.1 (21.3-30.5)	training	[4]
Krishna et al. (2012b)	100	po, tab, SD (fed)	16	50	31.4 (19-45)	n.r.	n.r.	26.1 (21.3-30.5)	training	[4]
Krishna et al. (2012b)	100	po, sus, SD (fasting)	16	50	31.4 (19-45)	n.r.	n.r.	26.1 (21.3-30.5)	test	[4]
Krishna et al. (2012b)	100	po, sus, SD (fed)	16	50	31.4 (19-45)	n.r.	n.r.	26.1 (21.3-30.5)	test	[4]
Ezzet et al. (2005)	800	po, sus, QD	18	100	36 (26-44)	81.9 (63.6-100)	n.r.	n.r.	training	[5]
Ezzet et al. (2005)	400	po, sus, BID	18	100	36 (26-44)	81.9 (63.6-100)	n.r.	n.r.	test	[5]
Ezzet et al. (2005)	60	po, sus, QID	18	100	36 (26-44)	81.9 (63.6-100)	n.r.	n.r.	test	[5]

Study	Dose [mg]	Treatment	n	Men [%]	Age [yrs]	Weight [kg]	Height [cm]	BMI [kg/m²]	Dataset	References
Vuletić et al. (2019)	400	po, sus, SD	20	75	34.4 (20-51)	n.r.	n.r.	24.7 (20.5-29.8)	test	[6]
Courtney et al. (2003)	200	po, sus SD (high-fat breakfast)	20	100	n.r. (22-45)	n.r.	n.r.	n.r.	training	[7]
Courtney et al. (2003)	200	po, sus SD (non-fat breakfast)	20	100	n.r. (22-45)	n.r.	n.r.	n.r.	training	[7]
Courtney et al. (2003)	200	po, sus SD (fasting)	20	100	n.r. (22-45)	n.r.	n.r.	n.r.	training	[7]

n: number of individuals per study, *n.r.:* not reported, *iv*: intravenous, *po*: per os, *SD*: single dose, *MD*: multiple doses, *tab*: tablet, *sus*: suspension, *QD*: once daily, *BID*: twice daily, *TID*: three times a day, *w/o*: without. Values in brackets given for age, weight, and height are minima and maxima, all po administrations were given to human subjects

1.2 Drug-dependent parameters

The drug dependent parameters used in the final posaconazole PBPK model are summarized in Table S2 below.

Table S2. Summary of the POS parameters used in the final PBPK model

Parameter	Unit	Value used in PBPK model	Literature value [Reference]	Description
MW	[g/mol]	700.80	700.8 [8]	Molecular weight
pK_a 1 [base]		3.60	3.6 [8]	First acid dissociation constant
pK_a 2 [base]		4.60	4.6 [8]	Second acid dissociation constant
fu_P [%]		2.00	2.00 [8]	Fraction unbound in plasma
logP		4.58[a]	4.6 [8]	Lipophilicity
Solubility (pH 6.5)	[10^{-3} mg/mL]	7.72a	70, 10.2, 0.98, 2.8 [8]	Solubility
Partition coefficients		Poulin & Theil	[9,10]	Calculation method cell to plasma coefficients
Cellular permeabilities		PKSim® Standard	[11].	Calculation method permeation across cell membranes
Specific intestinal permeability	[cm/min]	5.05×10^{-5}	1.18×10^{-4} [12,13]	For SUS simulations
Specific intestinal permeability	[cm/s]	4.80×10^{-5}		For DR-tablet simulations
k_{cat} UGT1A4	[1/min]	16.52	16.9±0.55 [14]	Katalytic rate constant UGT1A4
K_M UGT1A4	[µmol/L]	15.90	15.9±1.19 [14]	Michaelis-Menten constant UGT1A4

[a] Model parameters have been estimated through parameter optimization based on the plasma concentrations; -- Value not available

1.3 Model evaluation

1.3.1 Goodness-of-fit plots of predicted vs observed plasma concentrations

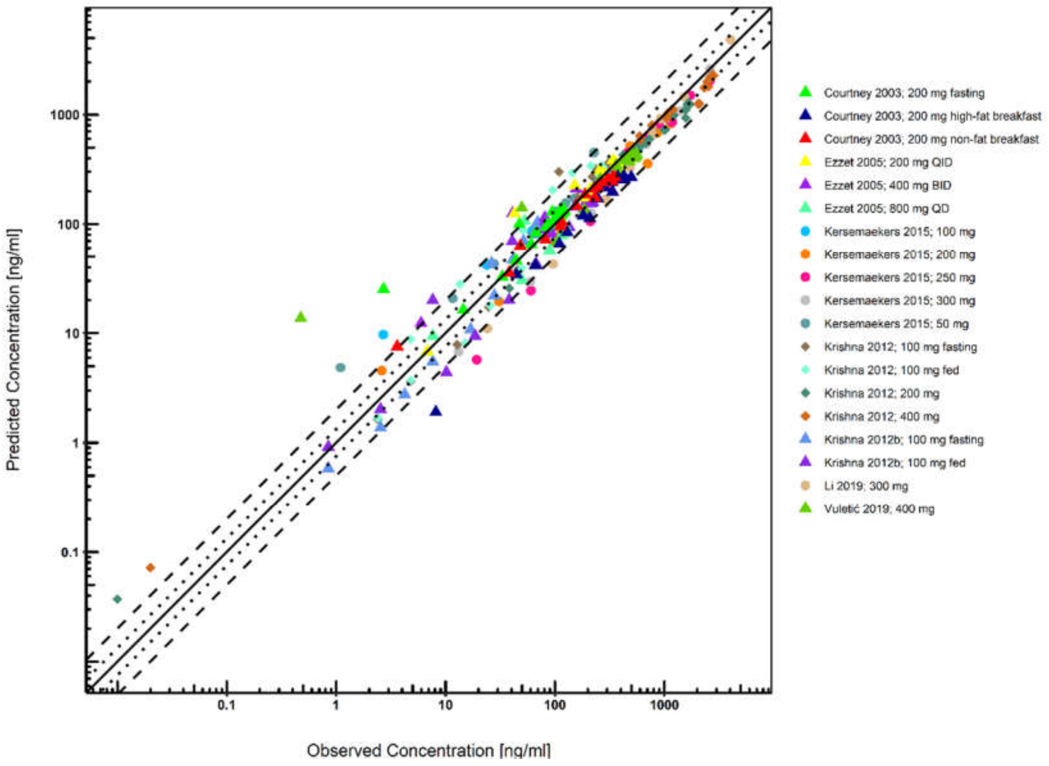

Figure S1. Predicted versus observed POS concentrations for i.v. (dots), DR-tablet (diamonds) and SUS (triangles). The black solid line marks the line of identity. Black dotted lines indicate 1.25-fold, black dashed lines indicate 2-fold deviation.

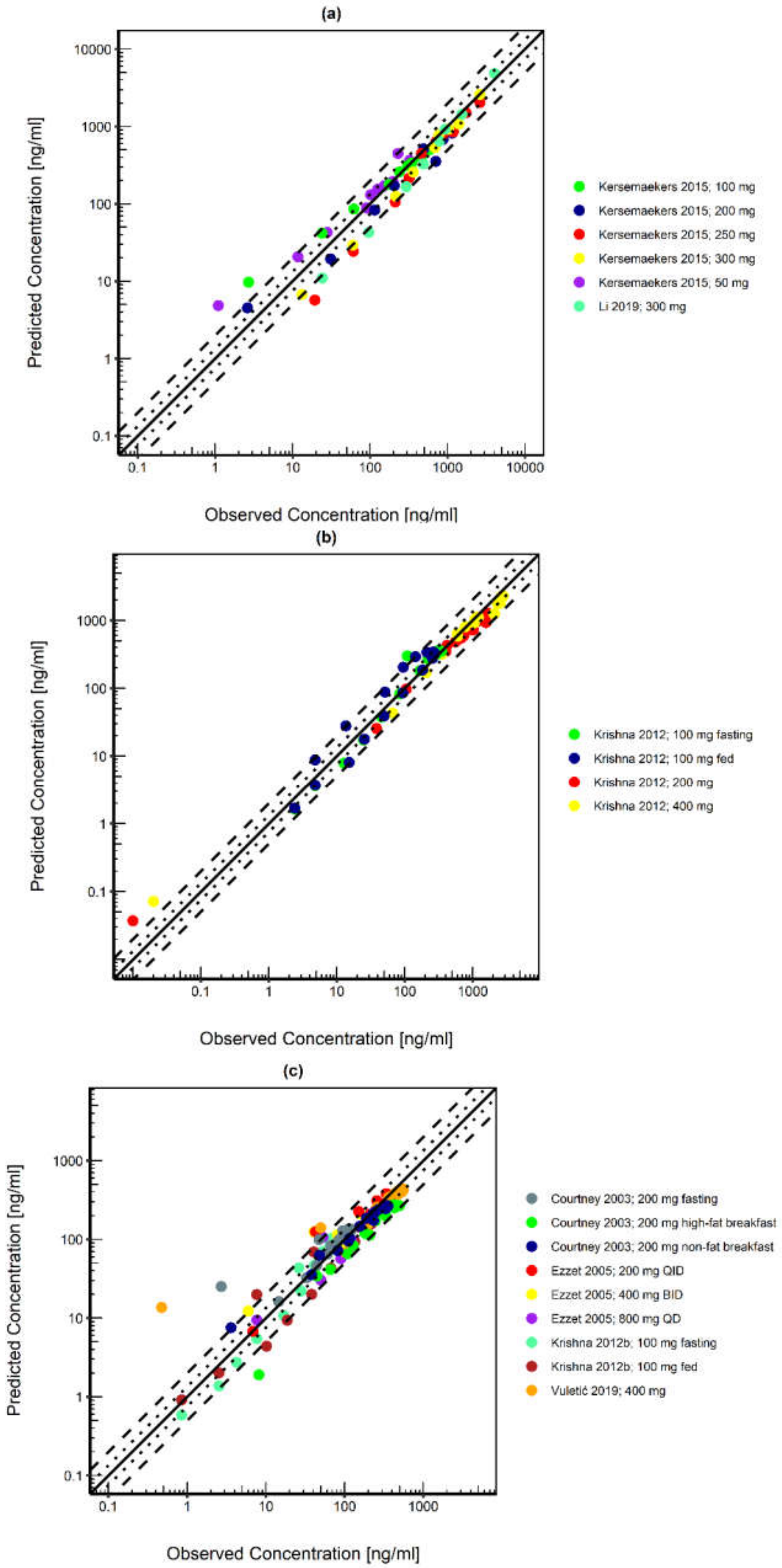

Figure S2. **(a)** **p**redicted versus observed POS concentrations for i.v. administration; **(b)** predicted versus observed POS concentrations for DR-tablet; **(c)** predicted versus observed POS concentrations for SUS administration The black solid line marks the line of identity. Black dotted lines indicate 1.25-fold, black dashed lines indicate 2-fold deviation.

1.3.2 Goodness of fit plot AUC_{last} and C_{max}

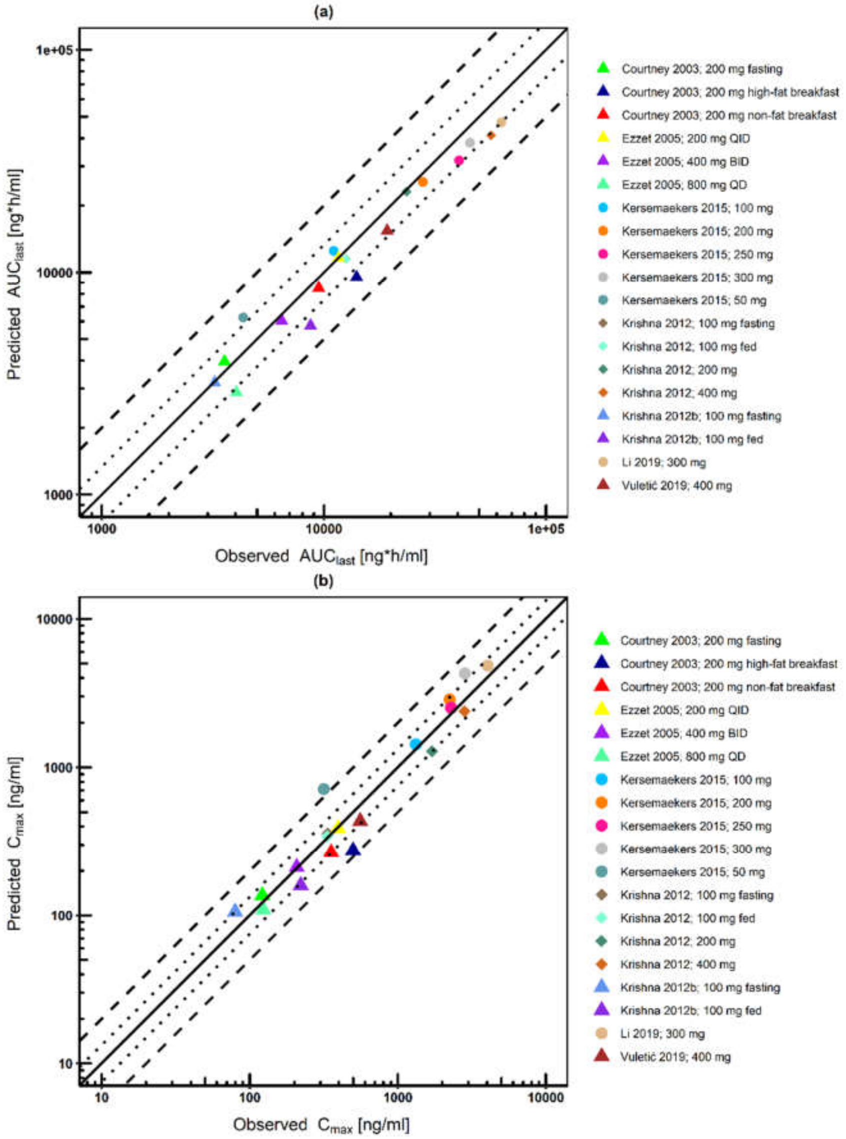

Figure S3. Predicted versus observed POS **(a)** AUC_{last} and **(b)** C_{max} values. Each symbol represents the AUC_{last} or C_{max} of a different profile. I.v. administrations are represented by diamonds, DR-tablet is represented by triangles, the SUS is represented by dots. The black solid line marks the line of identity. Black dotted lines indicate 1.25-fold, black dashed lines indicate 2-fold deviation. AUC_{last}: area under the plasma concentration–time curve from the time of administration to the last data point, C_{max}: maximum plasma concentration

1.3.3 Comparison of predicted and observed AUClast and Cmax

Table S3. Predicted and observed pharmacokinetic parameters of POS after intravenous and oral administration

Route, Dose	AUC_{last}			C_{max}			Reference
	Pred [ng*h/ml]	Obs [ng*h/ml]	Pred/Obs	Pred [ng/ml]	Obs [ng/ml]	Pred/Obs	
iv, 30 min, 50 mg	6246.8	4337.6	1.44	714.03	316.58	2.26	Kersemaekers et al. 2015 [1]
iv, 30 min, 100 mg	12495.58	11071.89	1.13	1428.07	1322.21	1.08	Kersemaekers et al. 2015 [1]
iv, 30 min, 200 mg	25488.31	27887.93	0.91	2856.20	2241.43	1.27	Kersemaekers et al. 2015 [1]
iv, 30 min, 250 mg	31852.54	40600.00	0.78	2519.31	2291.27	1.10	Kersemaekers et al. 2015 [1]
iv, 30 min, 300 mg	38243.1	45500.00	0.84	4284.4	2840.00	1.51	Kersemaekers et al. 2015 [1]
iv, 30 min, 300 mg	47181.13	62938.74	0.75	4846.02	4043.35	1.20	Li et al. 2019 [2]
oral, tab, MD, 200 mg	25593.18	23560.84-	1.09	1459.45	1705.64	0.86	Krishna et al. 2012 [3]
oral, tab, MD, 400 mg	52149.22	56581.78	0.92	2997.58	2829.40	1.06	Krishna et al. 2012 [3]
oral, tab (fasting), 100 mg	11473.41	12567.66	0.91	376.18	336.28	1.12	Krishna et al. 2012 [4]
oral, tab (fed), 100 mg	12518.61	12555.30	1.00	333.56	332.410	1.0	Krishna et al. 2012 [4]
oral, sus (fasting), 100 mg	3184.2	3224.18	0.99	105.6	79.66	1.33	Krishna et al. 2012 [4]
oral, sus (fed), 100 mg	5748.17	8706.51	0.66	159.5	221.19	0.72	Krishna et al. 2012 [4]
oral, sus, QD, 800 mg	2873.80	4038.40	0.71	108.9	123.62	0.88	Ezzet et al. 2005 [5]
oral, sus, BID, 400 mg	6050.90	6453.20	0.94	211.20	207.60	1.02	Ezzet et al. 2005 [5]
oral, sus, QID, 200 mg	11591.95	11625.10	1.00	386.10	394.32	0.98	Ezzet et al. 2005 [5]
oral, sus, 400 mg	15399.68	19261.75	0.80	435.00	557.35	0.78	Vuletic et al. 2019 [6]
oral, sus, (fasting), 200 mg	3962.51	3566.30	1.11	136.07	121.58	1.12	Courtney et al. 2003 [7]
oral, sus, (high fat breakfast), 200 mg	9523.2	14021.83	0.68	275.48	498.26	0.55	Courtney et al. 2003 [7]
oral, sus, (non-fat breakfast), 200 mg	8496.86	9474.29	0.90	266.48	354.85	0.75	Courtney et al. 2003 [7]

AUC_{last}: Area under the concentration time curve from the first to the last data point, *cap:* capsule, *C_{max}:* maximum plasma concentration, *Obs:* observed value, *Pred:* predicted value, iv: intravenous, *sus:* suspension, *tab:* tablet, *MD:* multiple dosing, *QD*: once daily, *BID*: twice daily; *TID:* three times a day

1.3.4 Bias, prediction and mean relative deviation of plasma predictions

Table S4. Bias (mean prediction error), precision (mean absolute prediction error) and mean relative deviation (MRD).

Route	Dose [mg]	MPE [%]	MAPE [%]	MRD	Reference
**Intravenous**					
iv, 30 min	50	33.98	34.38	1.42	Kersemaekers et al. 2015 [1]
iv, 30 min	100	39.60	46.50	1.63	Kersemaekers et al. 2015 [1]
iv, 30 min	200	-12.49	30.05	1.46	Kersemaekers et al. 2015 [1]
iv, 30 min	250	-29.65	29.65	1.75	Kersemaekers et al. 2015 [1]
iv, 30 min	300	-25.00	25.01	1.50	Kersemaekers et al. 2015 [1]
iv, 30 min	300	-23.55	28.52	1.61	Li et al. 2019 [2]
**mean MRD**				**1.56 (1.42 – 1.75)**	
				**6/6 with MRD ≤ 2**	
**Oral**					
oral, tablet	200	-11.84	16.02	1.24	Krishna et al. 2012 [3]
oral, tablet	400	3.70	14.67	1.19	Krishna et al. 2012 [3]
oral, tablet (fasting)	100	1.54	22.51	1.31	Krishna et al. 2012 [4]
oral, tablet (fed)	100	34.04	39.26	1.48	Krishna et al. 2012 [4]
oral, sus (fasting)	200	6.86	33.29	1.43	Krishna et al. 2012 [4]
oral, sus (fed)	200	-6.82	40.50	1.63	Krishna et al. 2012 [4]
oral, sus, QD	800	-11.24	33.87	1.47	Ezzet et al. 2005 [5]
oral, sus, BID	400	25.07	36.47	1.46	Ezzet et al. 2005 [5]
oral, sus, QID	200	10.22	17.83	1.30	Ezzet et al. 2005 [5]
oral, sus	400	147.64	181.61	2.36	Vuletic et al. 2019 [6]
oral, sus, (fasting)	200	66.57	66.72	1.80	Courtney et al. 2003 [7]
oral, sus, (high fat breakfast)	200	-36.85	36.85	1.75	Courtney et al. 2003 [7]
oral, sus, (non-fat breakfast)	200	-3.26	20.77	1.29	Courtney et al. 2003 [7]
**mean MRD**				**1.52 (1.19 – 2.36)**	
				**12/13 with MRD ≤ 2**	

iv: intravenous, *sus:* suspension; *tab:* tablet; *QD*: once daily; *BID*: twice daily; *TID*: three times a day; *MPE:* mean prediction error, *MAPE:* mean absolute prediction error, *MRD:* mean relative deviation

1.3.5 Sensitivity analysis

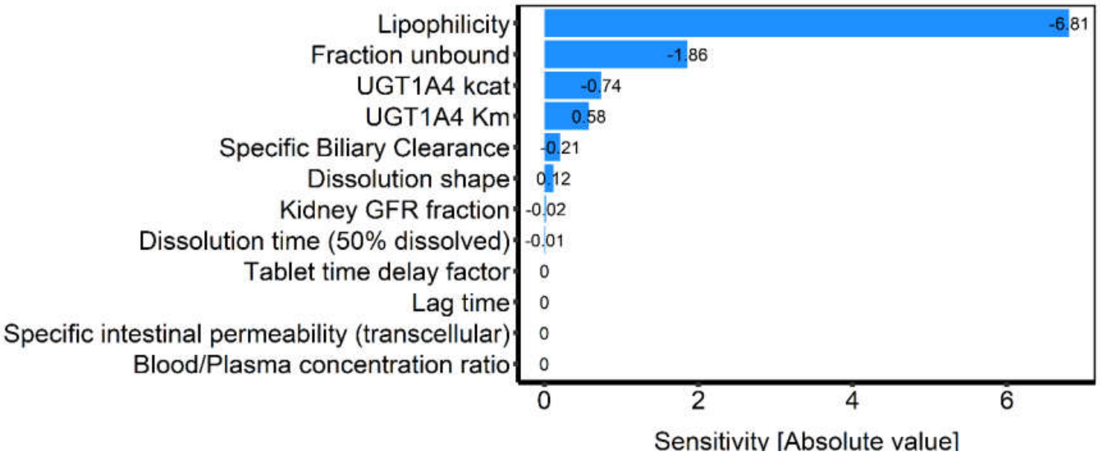

Figure S4. Sensitivity analysis for parameters which were estimated during the model development or which might have an impact due to calculation methods in PK-Sim®. Sensitivity was measured as the relative change of AUC_{last} of a 100 mg POS tablet single dose administration in fasted state. Variation range was 10.0 with maximum number of steps = 9.

1.3.6 Linear plots

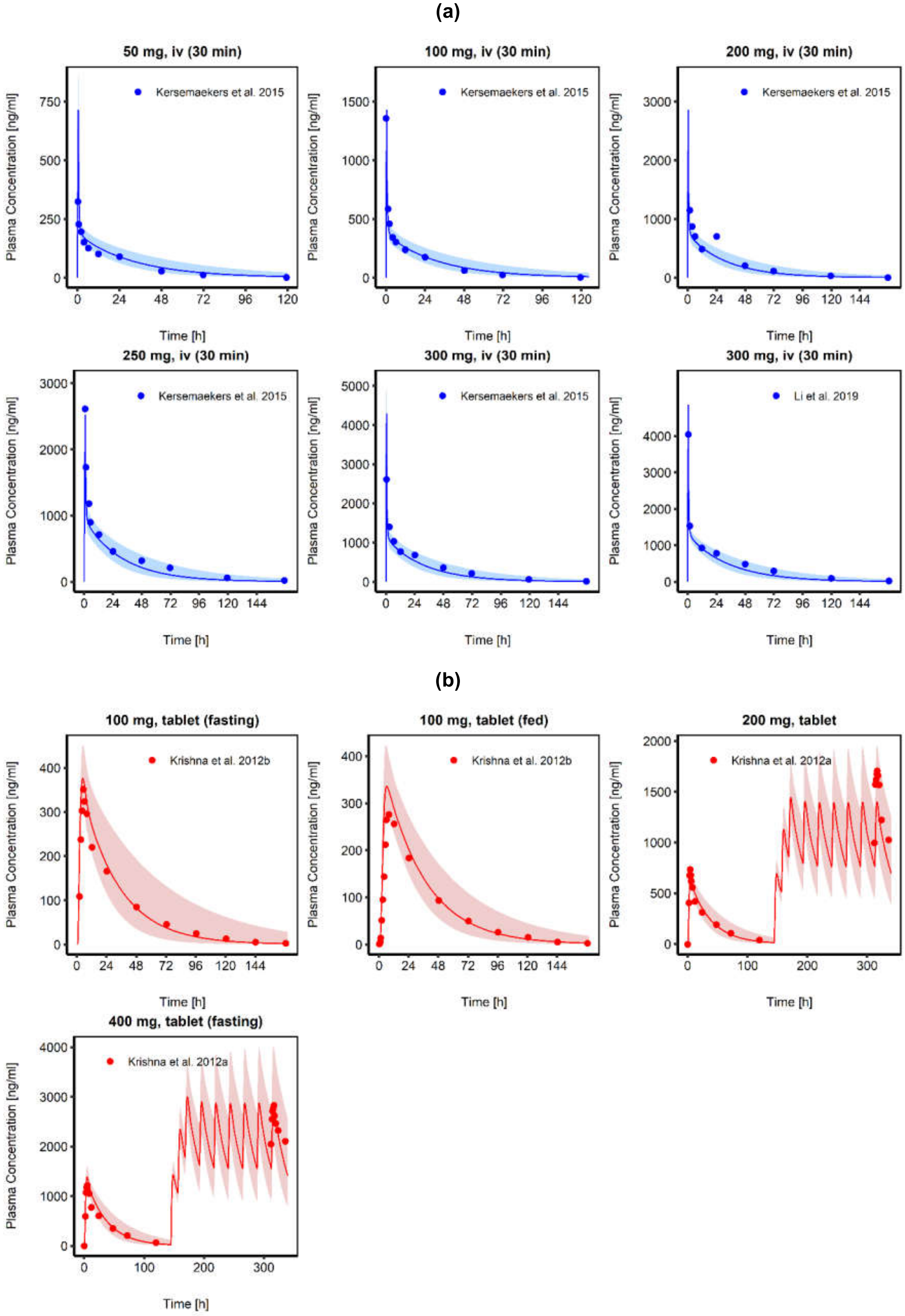

(c)

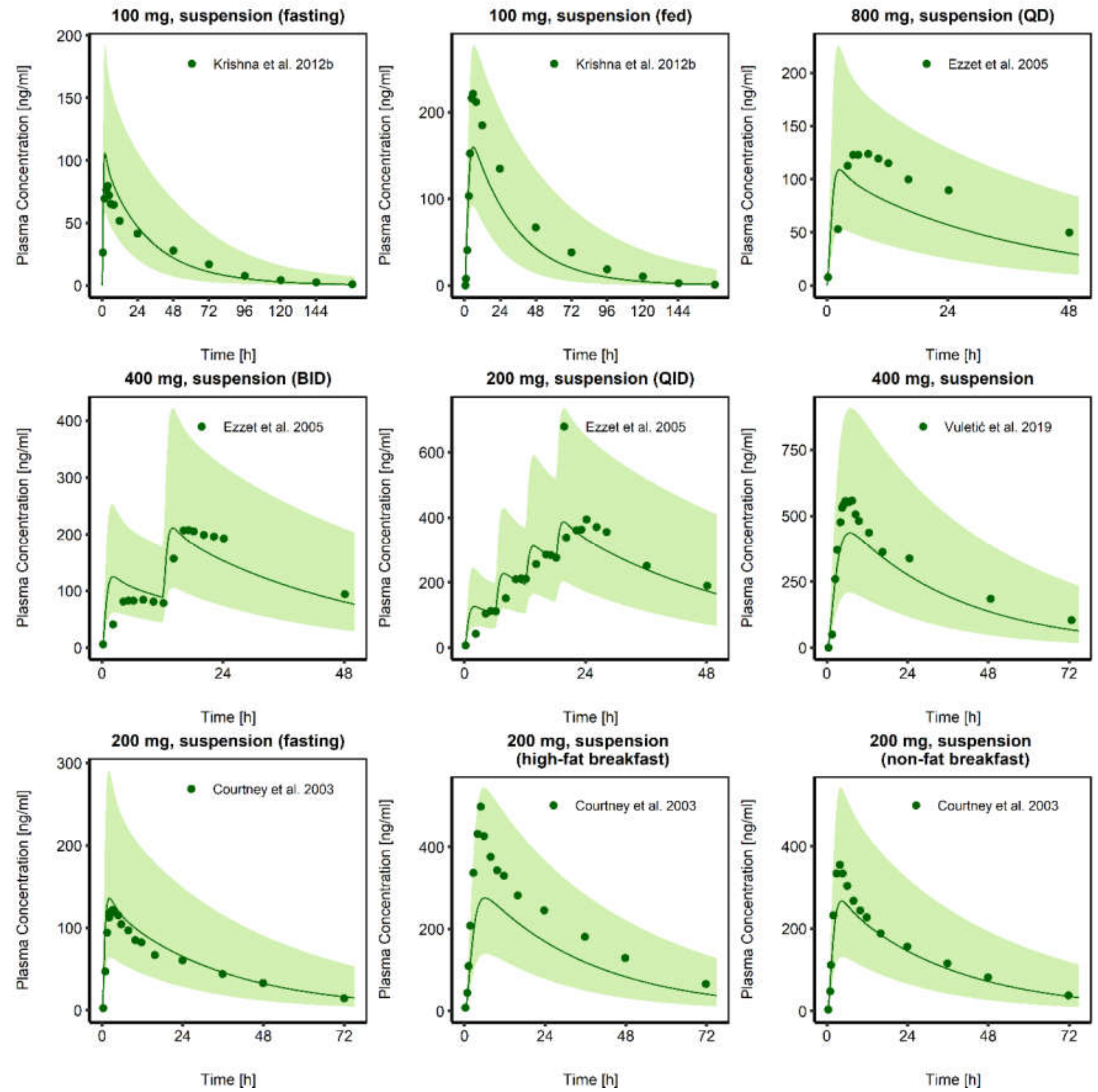

Figure S5. POS plasma concentration-time profiles (linear) after **(a)** i.v administration of POS, **(b)** POS DR-tablets and **(c)** POS SUS. Observed data are shown as blue (i.v.), red (DR-tablet) and green (SUS) circles. Population simulation (n=100) geometric means for each administration type are shown as blue, red and green lines, respectively. The shaded areas represent the predicted population geometric SD.

1.3.7 Semilogarithmic plots

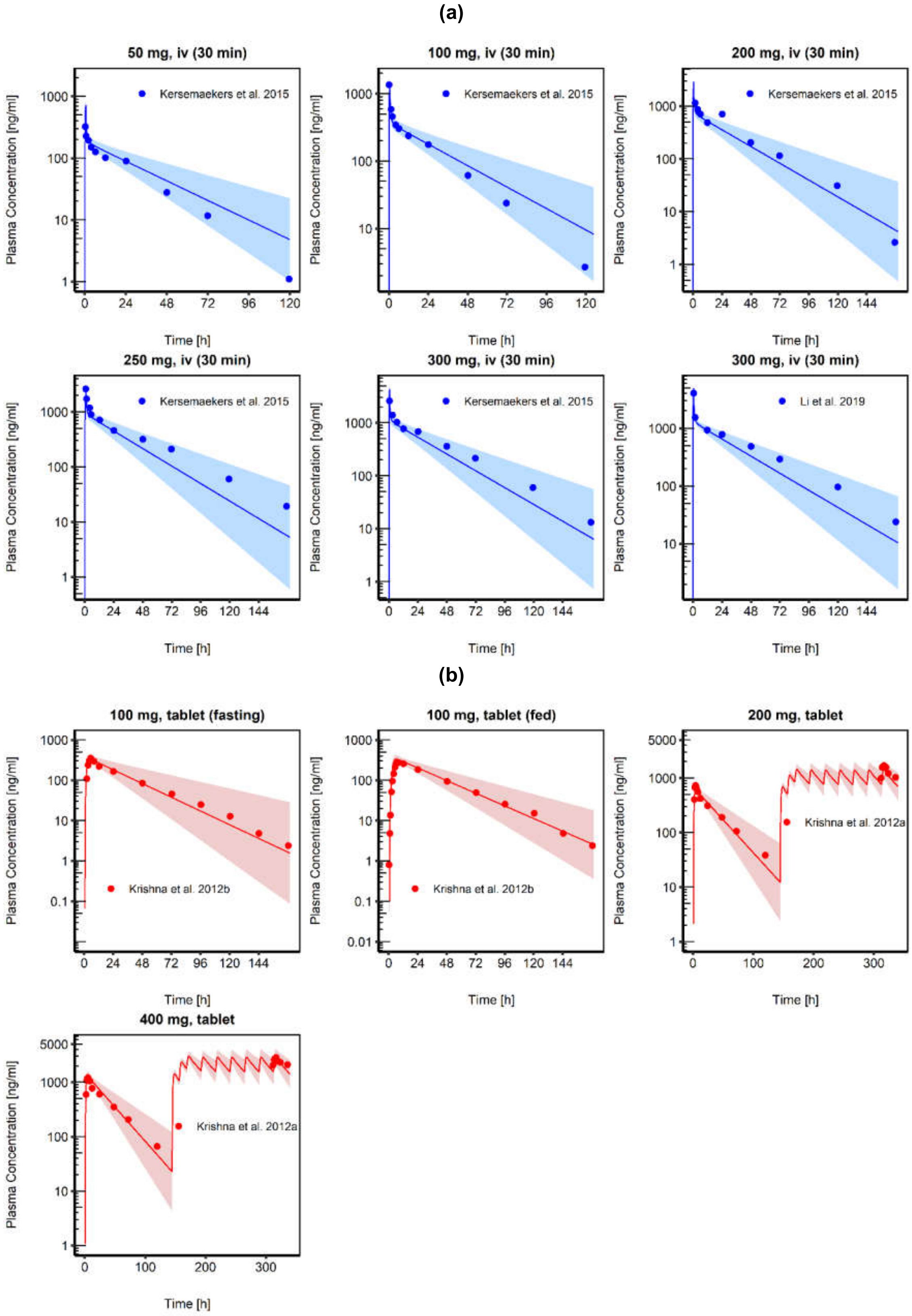

(c)

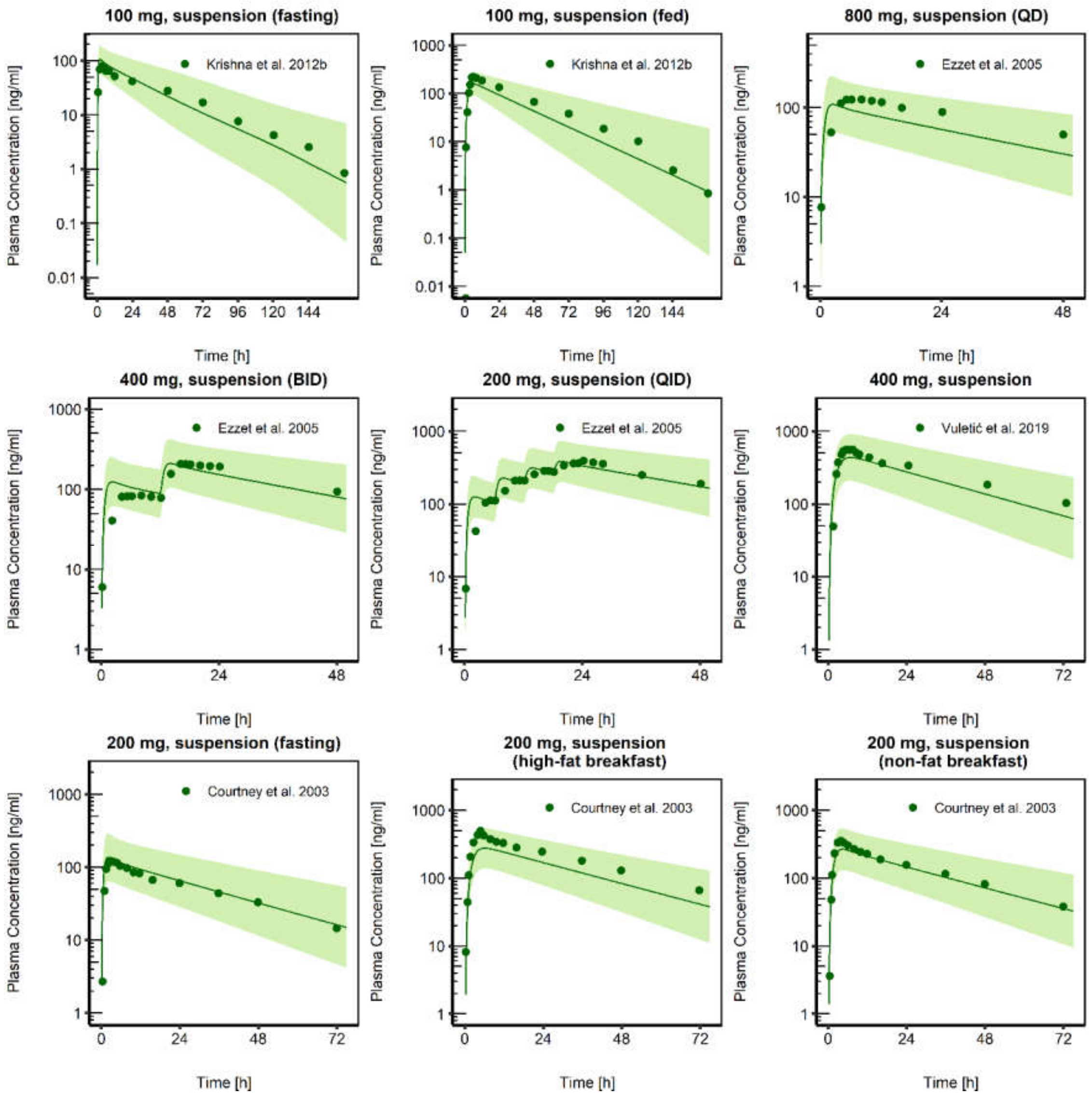

Figure S6. POS plasma concentration-time profiles (semi-logarithmic) after **(a)** i.v. administration of POS, **(b)** POS DR-tablets and **(c)** POS SUS. Observed data are shown as blue (i.v.), red (DR-tablet) and green (SUS) circles. Population simulation (n=100) geometric means for each administration type are shown as blue, red and green lines, respectively. The shaded areas represent the predicted population geometric SD.

1.3.8 Comparison of individual and population simulation

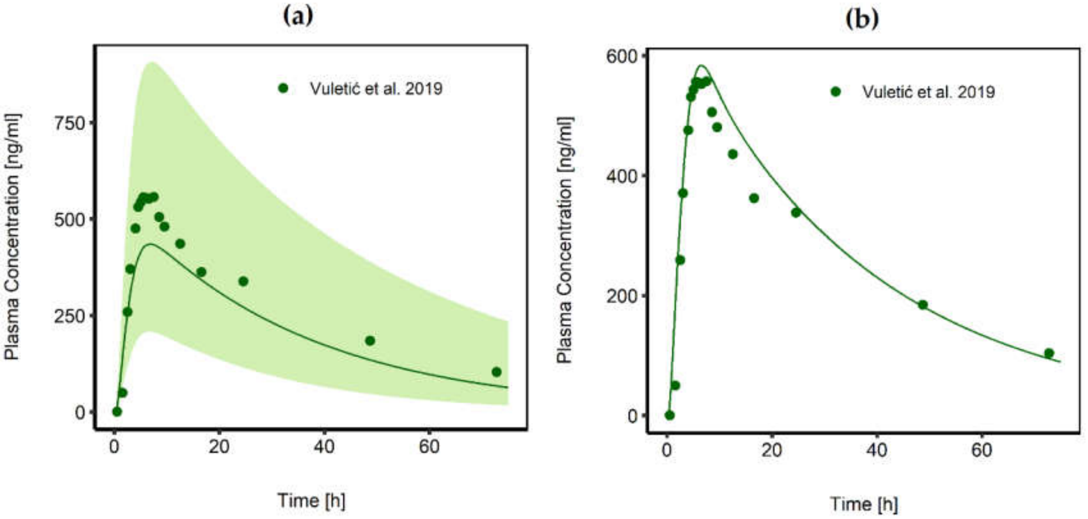

Figure S7. Comparison of predicted POS plasma concentration after administration of a 400 mg POS suspension single dose in **(a)** a virtual population (n = 100) created with the algorithm integrated in PKSim according to the patient demographics in the study conducted by Vuletic et al. [6] and predicted plasma concentration in **(b)** the mean induvial of the mentioned study. The green line represents the predicted geometric mean plasma concentration in the population respectively the predicted plasma concentration obtained for the individuum; the shaded area represents the geometric standard deviation for the population simulation; observed data are represented by green dots in each profile**.**

2 Ruxolitinib

2.1 Clinical studies

Table S5. Clinical studies used for the development of the Ruxolitinib PBPK model.

Study	Dose [mg]	Treatment	n	Men [%]	Age [yrs]	Weight [kg]	Height [cm]	BMI [kg/m²]	Dataset	References
Chen et al. (2014), hepatic	25	po, tab, SD	8	62.5	53 (45–59)	78.4 (61.8–93.2)	n.r.	27.5 (24.2–31.5)	test	[15]
Chen et al. (2014), renal	25	po, tab, SD	8	75	49 (22–69)	80.2 (64.7–89.8)	n.r.	26.9 (23.1–29.7)	test	[15]
Ogama et al. (2013)	10	po, tab, SD+MD	8	100	27 (20-41)	60.95 (52.5–75.9)	171.0 (159–182)	20.91 (18.8–24.5)	training	[16]
Ogama et al. (2013)	25	po, tab, SD+MD	8	100	27 (20-41)	60.95 (52.5–75.9)	171.0 (159–182)	20.91 (18.8–24.5)	training	[16]
Ogama et al. (2013)	50	po, tab, SD	8	100	27 (20-41)	60.95 (52.5–75.9)	171.0 (159–182)	20.91 (18.8–24.5)	training	[16]
Ogama et al. (2013)	100	po, tab, SD	8	100	27 (20-41)	60.95 (52.5–75.9)	171.0 (159–182)	20.91 (18.8–24.5)	training	[16]
Shi et al. (2011)	15	po, tab, BID, MD	71	77.5	29 (18-54)	75.1 (51.1-98.5)	n.r.	24.8 (19.8-29.6)	training	[17]
Shi et al. (2011)	25	po, tab, BID, MD	71	77.5	29 (18-54)	75.1 (51.1-98.5)	n.r.	24.8 (19.8-29.6)	test	[17]
Shi et al. (2011)	50	po, tab, QD, MD	71	77.5	29 (18-54)	75.1 (51.1-98.5)	n.r.	24.8 (19.8-29.6)	test	[17]
Shi et al. (2011)	50	po, tab, BID, MD	71	77.5	29 (18-54)	75.1 (51.1-98.5)	n.r.	24.8 (19.8-29.6)	test	[17]
Shi et al. (2011)	100	po, tab, QD, MD	71	77.5	29 (18-54)	75.1 (51.1-98.5)	n.r.	24.8 (19.8-29.6)	test	[17]

n: number of individuals per study, *n.r.:* not reported, *po*: per os, *SD*: single dose, *MD*: multiple doses, *QD*: once daily, *BID*: twice daily, *w/o*: without. Values in brackets given for age, weight, and height are minima and maxima,

2.2 Drug-dependent parameters

Table S6. Summary of the RUX parameters used in the final PBPK model

Parameter	Unit	Value used in PBPK model	Literature value [Reference]	Description
MW	[g/mol]	306.00	306.0 [18]	Molecular weight
pK_a [base]		3.89	3.89 [18]	Acid dissociation constant
fu_P [%]		3.30	3.30 [18]	Fraction unbound in plasma
logP		2.81	2.81 [18]	Lipophilicity
Solubility (pH 6.5)	[10^{-3} mg/mL]		0.3 [19]	Solubility
Partition coefficients		Rodgers & Rowland	[9,10]	Calculation method cell to plasma coefficients
Cellular permeabilities		PKSim® Standard	--	Calculation method permeation across cell membranes
Specific intestinal permeability	[10^{-4} cm/s]	5.40	5.4 [19]	
CYP 2C9 in vitro CL/recombinant enzyme	[µl/min/pmol rec. enzyme]	0.65	0.648 [18]	In vitro metabolic rate in the presence of CYP2C9
CYP 3A4 in vitro CL/recombinant enzyme	[µl/min/pmol rec. enzyme]	0.46	0.463 [18]	In vitro metabolic rate in the presence of CYP3A4
GFR Fraction		1.0	--	Fraction of filtered drug in the urine
Tablet Weibull time	[min]	15	--	Dissolution time (50 % dissolved)
Tablet Weibull shape		1.10	--	Dissolution profile shape

[a] model parameters have been estimated through parameter optimization based on the plasma concentrations; -- Value not available

2.3 Model evaluation

2.3.1 Goodness-of-fit plots of predicted vs observed plasma concentrations

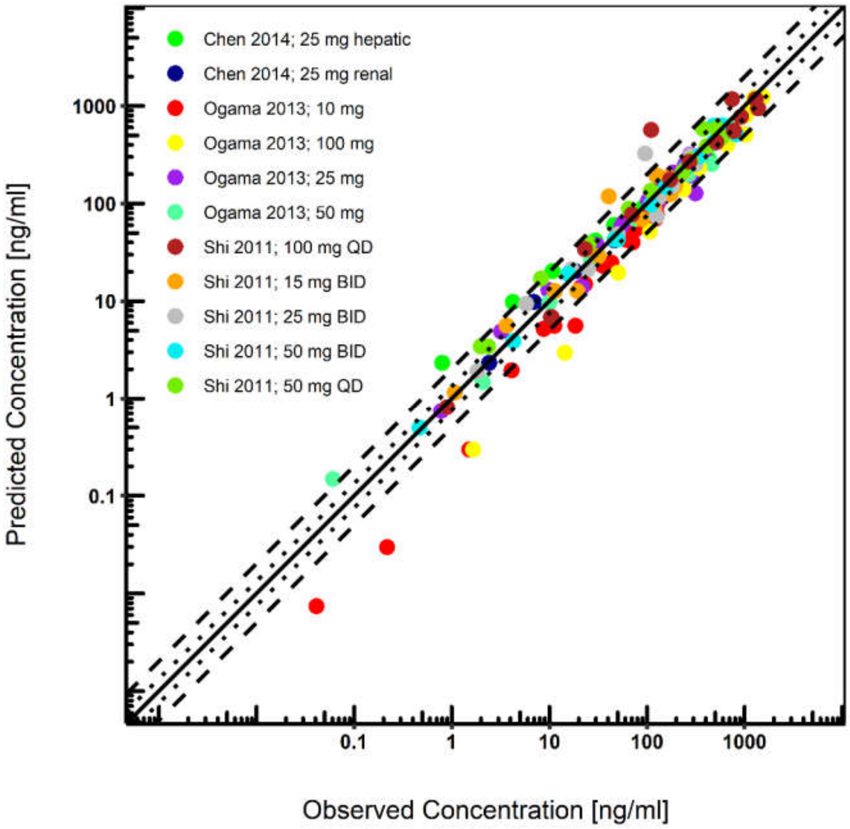

Figure S8. Predicted versus observed RUX concentrations after oral administration. Each dot represents measured plasma concentrations of the respective study The black solid line marks the line of identity. Black dotted lines indicate 1.25-fold, black dashed lines indicate 2-fold deviation.

2.3.2 AUC$_{last}$ and C$_{max}$ goodness-of-fit plots

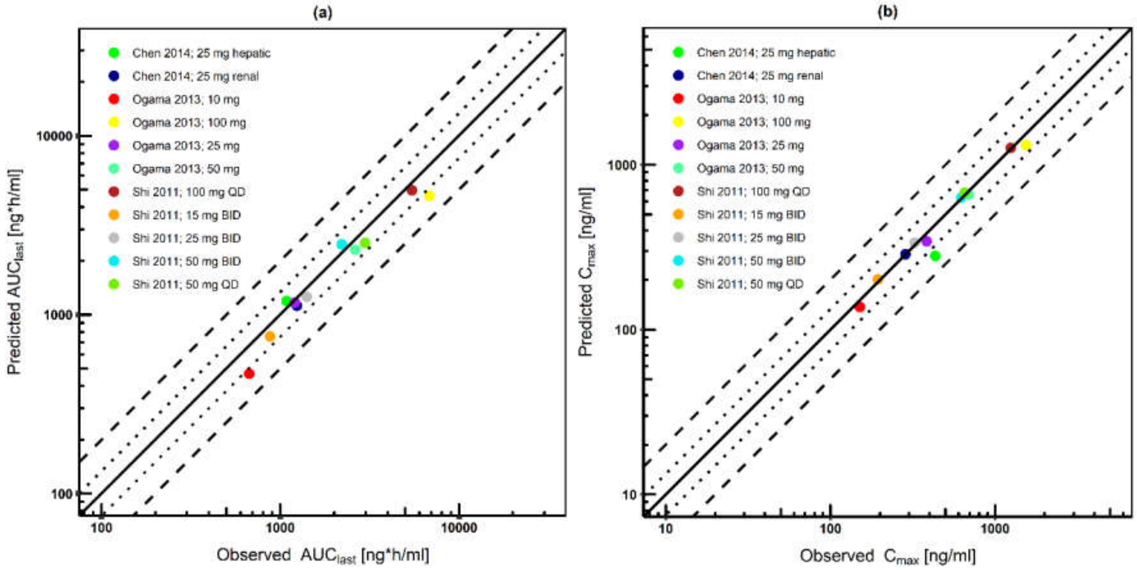

Figure S9. (a) Predicted versus observed ruxolitinib AUC$_{last}$. and **(b)** predicted versus observed ruxolitinib maximum concentration (C$_{max}$). Each symbol represents C$_{max}$ respectively AUC$_{last}$ of a different profile The black solid line marks the line of identity. Black dotted lines indicate 1.25-fold, black dashed lines indicate 2-fold deviation.

2.3.3 Comparison of predicted and observed AUClast and Cmax

Table S7. Predicted and observed pharmacokinetic parameters of RUX after oral administration

**Route, Dose**	**AUC_{last}**			**C_{max}**			**Reference**
	**Pred [ng*h/ml]**	**Obs [ng*h/ml]**	**Pred/Obs**	**Pred [ng/ml]**	**Obs [ng/ml]**	**Pred/Obs**	
po, tab, SD, 25 mg	1191.6	1085.92	1.10	279.95	433.93	0.65	Chen et al. (2014), hepatic [15]
po, tab, SD, 25 mg	1122.52	1237.86	0.91	286.28	285.44	1.00	Chen et al. (2014), renal [15]
po, tab, SD+MD, 10 mg	468.48	670.76	0.70	137.15	150.94	0.91	Ogama et al. (2013) [16]
po, tab, SD+MD, 25 mg	1171.21	1207.14	0.97	342.88	384.35	0.89	Ogama et al. (2013) [16]
po, tab, SD, 50 mg	2305.43	2632.77	0.88	657.33	695.28	0.95	Ogama et al. (2013) [16]
po, tab, SD, 100 mg	4610.86	6827.36	0.68	1314.66	1544.16	0.85	Ogama et al. (2013) [16]
po, tab, BID, MD, 15 mg	754.78	877.97	0.86	201.94	194.62	1.04	Shi et al. (2011) [17]
po, tab, BID, MD, 25 mg	1257.97	1406.70	0.89	336.56	324.37	1.04	Shi et al. (2011) [17]
po, tab, QD, MD, 50 mg	2471.90	2207.04	1.12	633.37	622.98	1.02	Shi et al. (2011) [17]
po, tab, BID, MD, 50 mg	2517.59	2983.86	0.84	677.41	654.45	1.04	Shi et al. (2011) [17]
po, tab, QD, MD, 100 mg	4944.03	5457.58	0.91	1265.54	1245.98	1.02	Shi et al. (2011) [17]

AUC_{last}: Area under the concentration time curve from the first to the last data point, *cap:* capsule, *C_{max}:* maximum plasma concentration, *Obs:* observed value, *Pred:* predicted value, *tab:* tablet, *SD:* single dose, *MD:* multiple dosing, *QD*: once daily, *BID*: twice daily

2.3.4 Bias, prediction and mean relative deviation of plasma predictions

Table S8. Bias (mean prediction error), precision (mean absolute prediction error) and mean relative deviation (MRD) of the RUX PBPK model.

Route	Dose [mg]	MPE	MAPE	MRD	Reference
po, tab, SD	25	33.29	54.81	1.68	Chen et al. (2014), hepatic [15]
po, tab, SD	25	-7.27	17.70	1.24	Chen et al. (2014), renal [15]
po, tab, SD+MD	10	-39.83	39.83	2.18	Ogama et al. (2013) [16]
po, tab, SD+MD	25	-0.19	17.96	1.32	Ogama et al. (2013) [16]
po, tab, SD	50	-1.21	24.72	1.40	Ogama et al. (2013) [16]
po, tab, SD	100	-40.92	40.92	2.23	Ogama et al. (2013) [16]
po, tab, BID, MD	15	14.77	37.18	1.49	Shi et al. (2011) [17]
po, tab, BID, MD	25	18.69	38.75	1.54	Shi et al. (2011) [17]
po, tab, QD, MD	50	31.23	36.82	1.43	Shi et al. (2011) [17]
po, tab, BID, MD	50	-4.72	14.30	1.20	Shi et al. (2011) [17]
po, tab, QD, MD	100	25.20	50.99	1.71	Shi et al. (2011) [17]
**mean MRD**				**1.58 (1.20 – 2.23)<br>09/11 with MRD ≤ 2**	

tab: tablet; *SD:* single dose, *MD*: multiple doses, *QD*: once daily; *BID*: twice daily; *MPE:* mean prediction error, *MAPE:* mean absolute prediction error, *MRD:* mean relative deviation

2.3.5 Sensitivity analysis

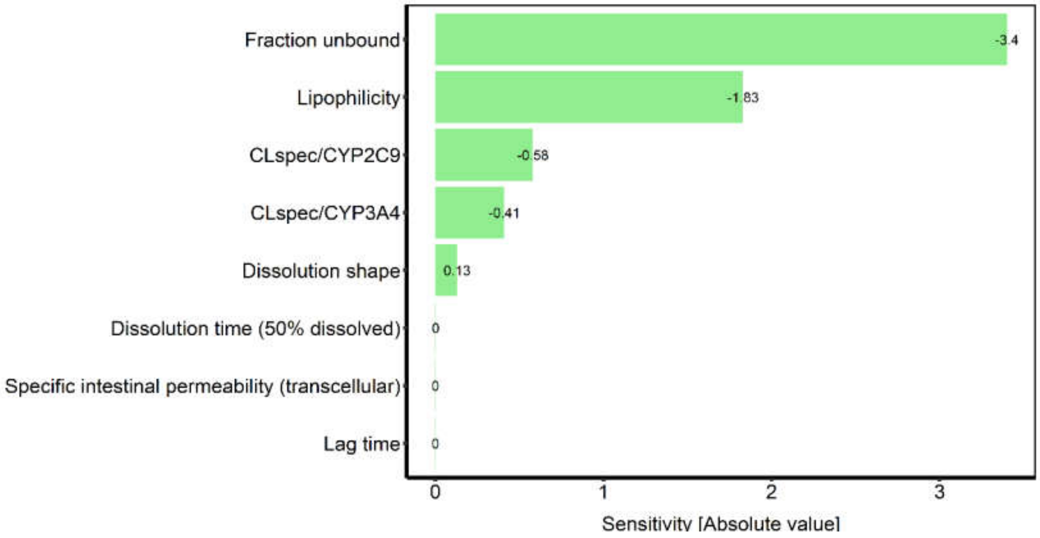

Figure S10. RUX sensitivity analysis for parameters which were estimated during the model development or which might have an impact due to calculation methods in PK-Sim®. Sensitivity was measured as the relative change of AUC_{last} of a 50 mg RUX BID tablet administration in fasted state. Variation range was 10.0 with maximum number of steps = 9.

2.3.6 Semilogarithmic plots

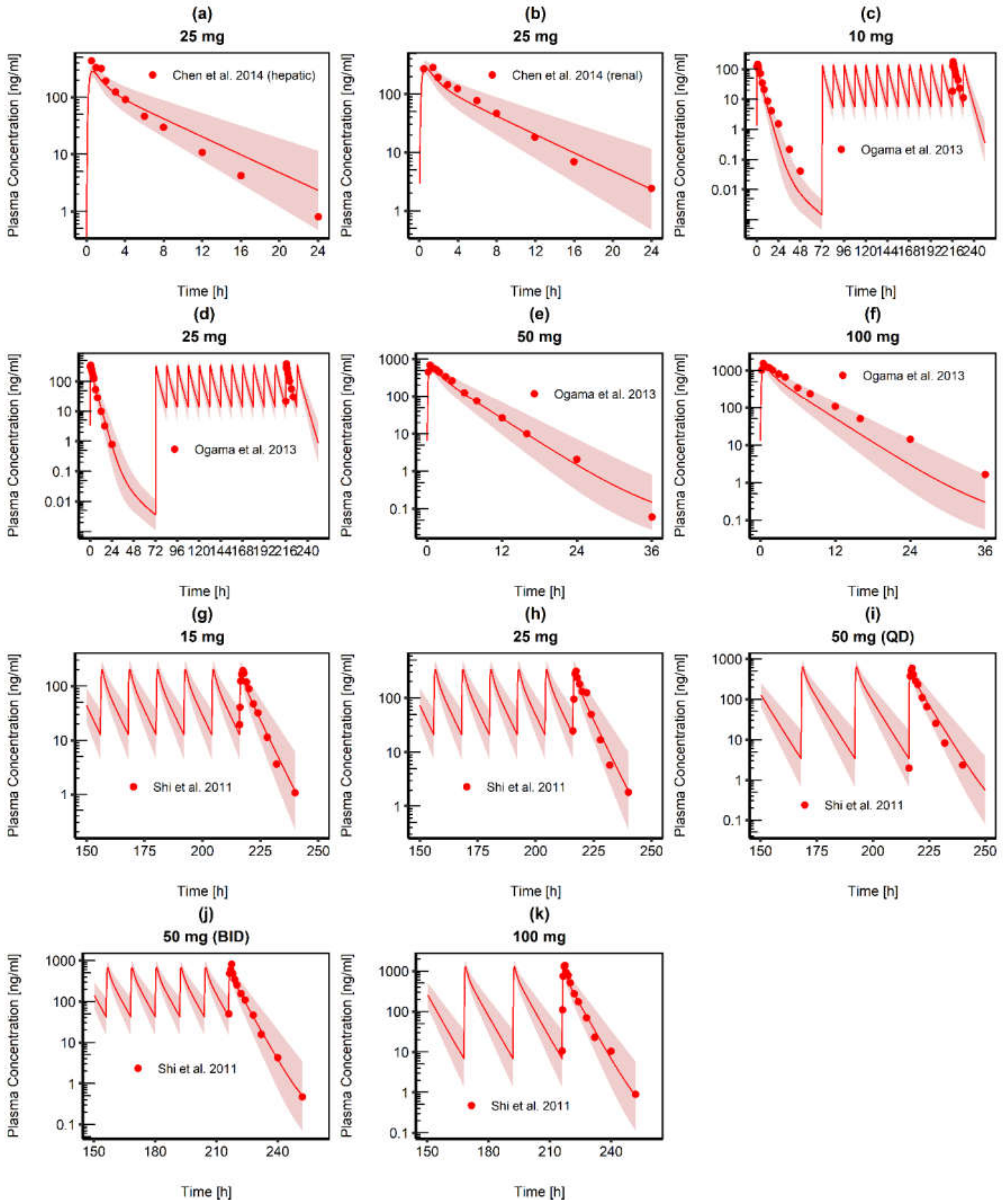

Figure S11. RUX plasma concentration-time profiles (semi-logarithmic) after administration of RUX tablet. Observed data are shown as red dots. Population simulation (n=100) geometric means are shown as red lines; the shaded areas represent the predicted population geometric SD.

3 Drug-drug interaction simulation posaconazole and midazolam

3.1 Clinical studies

Table S9. Clinical studies used for the investigation and evaluation of the inhibitory constant of POS for CYP3A4.

Study	Dose [mg]	MDZ Treatment	n	Men [%]	Age [yrs]	Weight [kg]	Height [cm]	BMI [kg/m²]	References
Krishna et al. (2009)	0.4 mg MDZ + 200 mg POS	i.v., 30 min	12	92	42.8 (28-53)	80.6 (69.4-94.9)	n.r.	25.6 (22.7-28.8)	[20]
Krishna et al. (2009)	0.4 mg MDZ + 400 mg POS	i.v., 30 min	12	92	42.8 (28-53)	80.6 (69.4-94.9)	n.r.	25.6 (22.7-28.8)	[20]
Krishna et al. (2009)	0.4 mg MDZ	i.v., 30 min	12	92	42.8 (28-53)	80.6 (69.4-94.9)	n.r.	25.6 (22.7-28.8)	[20]
Krishna et al. (2009)	2 mg MDZ + 200 mg POS	oral	12	92	42.8 (28-53)	80.6 (69.4-94.9)	n.r.	25.6 (22.7-28.8)	[20]
Krishna et al. (2009)	2 mg MDZ + 200 mg POS	oral	12	92	42.8 (28-53)	80.6 (69.4-94.9)	n.r.	25.6 (22.7-28.8)	[20]

4 Simulation of graft-versus-host disease patients

Table S10. Baseline patient demographics

Patient characteristic	No. of patients	%
Total	24	
Age [yrs], mean (range)	53 (22–80)	
Weight [kg], mean (range)	73.8 (43.0–111.0)	
Height [cm], mean, (range)	174 (156-196)	
BMI [kg/m^2], mean (range)	24.4 (16.2–43.4)	
Male	13	54.2
Female	11	45.8
RUX with POS	19[a]	79.2
RUX without POS	7[a]	29.2

[a] two patients were treated with RUX alone and in combination with POS

5 References

1. Kersemaekers, W.M.; van Iersel, T.; Nassander, U.; O'Mara, E.; Waskin, H.; Caceres, M.; van Iersel, M.L. Pharmacokinetics and safety study of posaconazole intravenous solution administered peripherally to healthy subjects. *Antimicrob. Agents Chemother.* **2015**, *59*, 1246-1251, doi:10.1128/AAC.04223-14.
2. Li, H.; Wei, Y.; Zhang, S.; Xu, L.; Jiang, J.; Qiu, Y.; Mangin, E.; Zhao, X.M.; Xie, S. Pharmacokinetics and Safety of Posaconazole Administered by Intravenous Solution and Oral Tablet in Healthy Chinese Subjects and Effect of Food on Tablet Bioavailability. *Clin. Drug. Investig.* **2019**, *39*, 1109-1116, doi:10.1007/s40261-019-00833-1.
3. Krishna, G.; Ma, L.; Martinho, M.; Preston, R.; O'mara, E. A new solid oral tablet formulation of posaconazole: a randomized clinical trial to investigate rising single-and multiple-dose pharmacokinetics and safety in healthy volunteers. *J. Antimicrob. Chemother.* **2012**, *67*, 2725-2730.
4. Krishna, G.; Ma, L.; Martinho, M.; O'Mara, E. Single-dose phase I study to evaluate the pharmacokinetics of posaconazole in new tablet and capsule formulations relative to oral suspension. *Antimicrob. Agents Chemother.* **2012**, *56*, 4196-4201.
5. Ezzet, F.; Wexler, D.; Courtney, R.; Krishna, G.; Lim, J.; Laughlin, M. Oral bioavailability of posaconazole in fasted healthy subjects. *Clin. Pharmacokinet.* **2005**, *44*, 211-220.
6. Vuletić, L.; Herceg, M.; Ferderber, K.; Tunjić, I.; Rizea-Savu, S.; Duna, S.N.; Cetina-Čižmek, B.; Filipović-Grčić, J. Single-Dose Pharmacokinetic Properties and Relative Bioavailability of Different Formulations of Posaconazole Oral Suspension in Healthy Volunteers. *Clin. Pharmacol. Drug Dev.* **2019**, *8*, 827-836.
7. Courtney, R.; Wexler, D.; Radwanski, E.; Lim, J.; Laughlin, M. Effect of food on the relative bioavailability of two oral formulations of posaconazole in healthy adults. *Br. J. Clin. Pharmacol.* **2004**, *57*, 218-222.
8. Hens, B.; Pathak, S.M.; Mitra, A.; Patel, N.; Liu, B.; Patel, S.; Jamei, M.; Brouwers, J.; Augustijns, P.; Turner, D.B. In Silico Modeling Approach for the Evaluation of Gastrointestinal Dissolution, Supersaturation, and Precipitation of Posaconazole. *Mol Pharm* **2017**, *14*, 4321-4333, doi:10.1021/acs.molpharmaceut.7b00396.

9. Rodgers, T.; Leahy, D.; Rowland, M. Physiologically based pharmacokinetic modeling 1: predicting the tissue distribution of moderate-to-strong bases. *Journal of pharmaceutical sciences* **2005**, *94*, 1259-1276.
10. Rodgers, T.; Rowland, M. Physiologically based pharmacokinetic modelling 2: predicting the tissue distribution of acids, very weak bases, neutrals and zwitterions. *Journal of pharmaceutical sciences* **2006**, *95*, 1238-1257.
11. Open Systems Pharmacology. PK-Sim®. Version 11.0. Available online: https://github.com/Open-Systems-Pharmacology/Suite/releases/tag/v11.0 (accessed on 2022 May 01).
12. Thelen, K.; Coboeken, K.; Willmann, S.; Dressman, J.B.; Lippert, J. Evolution of a detailed physiological model to simulate the gastrointestinal transit and absorption process in humans, part II: extension to describe performance of solid dosage forms. *Journal of pharmaceutical sciences* **2012**, *101*, 1267-1280.
13. Thelen, K.; Coboeken, K.; Willmann, S.; Burghaus, R.; Dressman, J.B.; Lippert, J. Evolution of a detailed physiological model to simulate the gastrointestinal transit and absorption process in humans, part 1: oral solutions. *Journal of pharmaceutical sciences* **2011**, *100*, 5324-5345.
14. Ghosal, A.; Hapangama, N.; Yuan, Y.; Achanfuo-Yeboah, J.; Iannucci, R.; Chowdhury, S.; Alton, K.; Patrick, J.E.; Zbaida, S. Identification of human UDP-glucuronosyltransferase enzyme(s) responsible for the glucuronidation of posaconazole (Noxafil). *Drug Metab Dispos* **2004**, *32*, 267-271, doi:10.1124/dmd.32.2.267.
15. Chen, X.; Shi, J.G.; Emm, T.; Scherle, P.A.; McGee, R.F.; Lo, Y.; Landman, R.R.; Punwani, N.G.; Williams, W.V.; Yeleswaram, S. Pharmacokinetics and pharmacodynamics of orally administered ruxolitinib (INCB018424 phosphate) in renal and hepatic impairment patients. *Clin. Pharmacol. Drug Dev.* **2014**, *3*, 34-42.
16. Ogama, Y.; Mineyama, T.; Yamamoto, A.; Woo, M.; Shimada, N.; Amagasaki, T.; Natsume, K. A randomized dose-escalation study to assess the safety, tolerability, and pharmacokinetics of ruxolitinib (INC424) in healthy Japanese volunteers. *Int. J. Hematol.* **2013**, *97*, 351-359.
17. Shi, J.G.; Chen, X.; McGee, R.F.; Landman, R.R.; Emm, T.; Lo, Y.; Scherle, P.A.; Punwani, N.G.; Williams, W.V.; Yeleswaram, S. The pharmacokinetics, pharmacodynamics, and safety of orally dosed INCB018424 phosphate in healthy volunteers. *J. Clin. Pharmacol.* **2011**, *51*, 1644-1654.
18. Umehara, K.; Huth, F.; Jin, Y.; Schiller, H.; Aslanis, V.; Heimbach, T.; He, H. Drug-drug interaction (DDI) assessments of ruxolitinib, a dual substrate of CYP3A4 and CYP2C9, using a verified physiologically based pharmacokinetic (PBPK) model to support regulatory submissions. *Drug Metab Pers Ther* **2019**, *34*, doi:10.1515/dmpt-2018-0042.
19. Shi, J.G.; Fraczkiewicz, G.; Williams, W.V.; Yeleswaram, S. Predicting drug-drug interactions involving multiple mechanisms using physiologically based pharmacokinetic modeling: a case study with ruxolitinib. *Clin Pharmacol Ther* **2015**, *97*, 177-185, doi:10.1002/cpt.30.
20. Krishna, G.; Moton, A.; Ma, L.; Savant, I.; Martinho, M.; Seiberling, M.; McLeod, J. Effects of oral posaconazole on the pharmacokinetic properties of oral and intravenous midazolam: a phase I, randomized, open-label, crossover study in healthy volunteers. *Clin. Ther.* **2009**, *31*, 286-298.
